# Recyclable hypervalent-iodine-mediated solid-phase peptide synthesis and cyclic peptide synthesis

**DOI:** 10.3762/bjoc.14.97

**Published:** 2018-05-22

**Authors:** Dan Liu, Ya-Li Guo, Jin Qu, Chi Zhang

**Affiliations:** 1 State Key Laboratory of Elemento-Organic Chemistry, Collaborative Innovation Center of Chemical Science and Engineering (Tianjin), College of Chemistry, Nankai University, Tianjin 300071, China

**Keywords:** cyclic peptide, FPID, hypervalent iodine(III) reagent, recyclable, solid-phase peptide synthesis (SPPS)

## Abstract

The system of the hypervalent iodine(III) reagent FPID and (4-MeOC_6_H_4_)_3_P was successfully applied to solid-phase peptide synthesis and cyclic peptide synthesis. Four peptides with biological activities were synthesized through SPPS and the bioactive cyclic heptapeptide pseudostellarin D was obtained via solution-phase peptide synthesis. It is worth noting that FPID can be readily regenerated after the peptide coupling reaction.

## Introduction

The amide bond is one of the most fundamental functional groups in organic chemistry, and it plays a crucial role in the elaboration and composition of biological systems. Amide bonds are widely present not only in peptides and proteins but also in pharmaceuticals and many natural products. Among the methods for amide bond formation, the direct condensation of carboxylic acids and amines in the presence of a coupling reagent is the most convenient and simplest way [1–6]. The most commonly used coupling reagents such as carbodiimide [7], phosphonium [8], and uronium salts [9] are efficient and commercially available. In spite of these merits of traditional coupling reagents, it is still far from ideal because large amounts of chemical wastes are produced during the amide bond formation reaction using these reagents and the coupling reagents cannot be regenerated [10]. Thus, methods for the peptide synthesis which are efficient and atom-economic are still needed.

**Figure 1:**
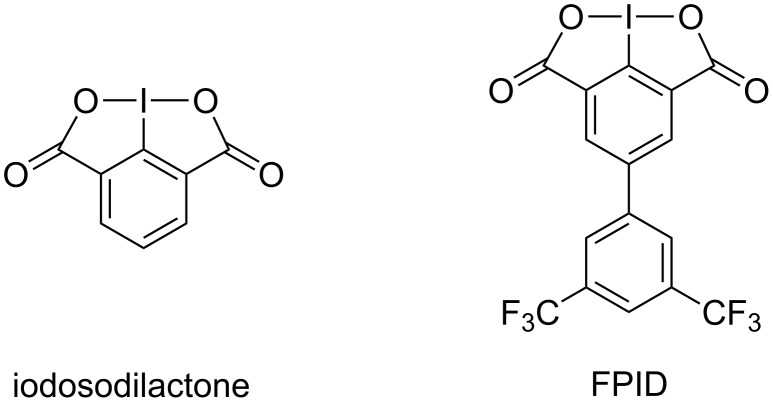
Iodosodilactone and FPID.

Hypervalent iodine reagents have drawn researchers’ considerable attentions due to their versatile reactivity, low toxicity, ready availability, environmental friendliness, and regenerability [11–27]. Our group has dedicated to the peptide synthesis mediated by hypervalent iodine(III) reagents in recent years. In 2012, for the first time, we reported that the hypervalent iodine(III) reagent iodosodilactone (Figure 1) can serve as a condensing reagent to promote esterification, macrolactonization, amidation and peptide coupling reactions in the presence of PPh_3_ [28]. In addition, the peptide coupling reaction proceeds without racemization in the absence of a racemization suppressant and iodosodilactone can be readily regenerated after the reaction. In order to further enhance the reactivity of iodosodilactone, we designed and synthesized a new derivative of iodosodilactone 6-(3,5-bis(trifluoromethyl)phenyl)-1*H*,4*H*-2aλ^3^-ioda-2,3-dioxacyclopenta[*hi*]indene-1,4-dione (abbreviated as FPID, Figure 1) [29].

**Scheme 1:**
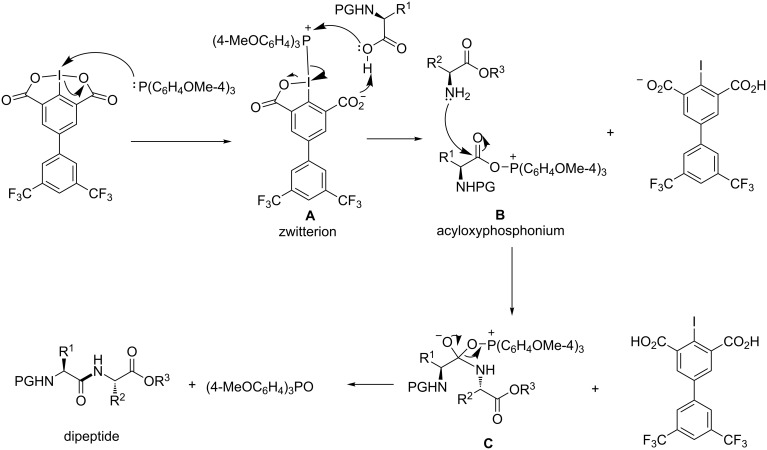
Proposed mechanism for FPID-mediated amide bond formation.

In combination with tris(4-methoxyphenyl)phosphine [(4-MeOC_6_H_4_)_3_P], FPID can efficiently mediate peptide coupling reactions within 30 minutes to obtain various dipeptides from standard amino acids as well as sterically hindered amino acids. Moreover, a pentapeptide Leu-enkephalin is successfully synthesized in its protected form using this coupling system. Similar to iodosodilactone, FPID can be easily regenerated after the reaction. The mechanism for this FPID-mediated amide bond formation reaction was proposed with the acyloxyphosphonium intermediate **B** being the key intermediate (Scheme 1). Herein, as part of our continuing exploration of the application of FPID in peptide synthesis, we disclose its successful application in solid-phase peptide synthesis and cyclic peptide synthesis.

## Results and Discussion

At the beginning of our study, we tried to utilize the system of FPID/(4-MeOC_6_H_4_)_3_P in the solid-phase peptide synthesis (SPPS). SPPS has been widely employed in peptide synthesis since its first report by Merrifield in 1963 [30–31]. Compared with classical solution-phase peptide synthesis, the fast development of SPPS is mainly due to its short reaction time, high efficiency, low racemization, simple work-up and automation. In recent decades, various strategies, for example, native chemical ligation (NCL) [32] and serine/threonine ligation (STL) [33], have been reported to solve the problems occurred during the development of SPPS. Peptide synthesis in solution mediated by FPID/(4-MeOC_6_H_4_)_3_P is rapid (within 30 min) and efficient, at the same time the reactions proceed without racemization. Thus, it is possible and significant to test whether the FPID/(4-MeOC_6_H_4_)_3_P system can be used in SPPS.

**Scheme 2:**
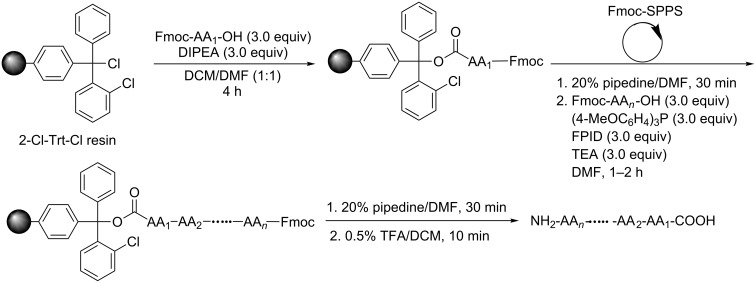
Solid-phase peptide synthesis mediated by FPID/(4-MeOC_6_H_4_)_3_P. Conditions: The resin loading for 2-Cl-Trt-Cl resin is 0.98 mmol/g. For each peptide synthesized through SPPS, 200 mg 2-Cl-Trt-Cl resin was used. Fmoc-Ser-OH, Fmoc-Thr-OH, Fmoc-Tyr-OH, Fmoc-Trp-OH were directly used without any protecting group on OH or NH. During the synthesis of **3**, Fmoc-Lys(Boc)-OH was used.

We selected the commercially available 2-chlorotrityl chloride resin (2-Cl-Trt-Cl resin) as the solid support and [(9-fluorenylmethyl)oxy]carbonyl (Fmoc) as the α-amino protecting group. The peptides were synthesized following the route as shown in Scheme 2. The C-terminal amino acid was immobilized onto the 2-Cl-Trt-Cl resin in the presence of 3.0 equiv of DIPEA in DCM/DMF (v:v 1:1). Subsequent peptide chain elongation was completed via Fmoc-SPPS protocol, which includes deprotection with 20% piperidine/DMF and peptide coupling with 3.0 equiv of Fmoc-protected amino acids, 3.0 equiv of FPID, 3.0 equiv of (4-MeOC_6_H_4_)_3_P and 3.0 equiv of TEA in DMF. After chain elongation and deprotection of Fmoc, the resulting resins were treated with 0.5% TFA/DCM to give the N,C-unprotected peptides as final products. The peptides were purified by reversed-phase HPLC (RP-HPLC).

For the target peptides, we aimed at peptides with specific biological activities. Leu-enkephalin, which is isolated from pig brains, acts as an endogenous mediator at central morphine receptor sites and thus possesses potent opiate agonist activity [34–36]. Leu-enkephalin **1** (Table 1, entry 1) could be successfully synthesized following the route mentioned above (Scheme 2). Besides, the precursor **2** of a cyclic heptapeptide pseudostellarin D [37–39] was also obtained via SPPS in good yield (Table 1, entry 2), the cyclization of **2** to give pseudostellarin D using FPID/(4-MeOC_6_H_4_)_3_P will be described in the following part. Moreover, angiotensin I converting enzyme (ACE) inhibitory peptides, widely exist in plants and animals, can serve as potential antihypertensive pharmaceuticals [40–42]. The synthesis of two ACE inhibitory peptides proceeded smoothly in moderate yield (Table 1, entries 3 and 4). Notably, it is unnecessary to protect the hydroxy group of serine, threonine, or tyrosine in advance in the synthesis of these four peptides. The presence of an unprotected hydroxy group does not affect the coupling efficiency, which is consistent with peptide coupling in solution phase [29]. The HRMS spectra of these peptides are consistent with their molecular formula.

**Table 1:** Peptides synthesized by SPPS mediated by FPID.

entry	peptide	yield

1	H_2_N-Tyr-Gly-Gly-Phe-Leu-OH (**1**)	42%
2	H_2_N-Gly-Gly-Tyr-Pro-Leu-Ile-Leu-OH (**2**)	53%
3	H_2_N-Lys-Leu-Pro-Ala-Gly-Thr-Leu-Phe-OH (**3**)	30%
4	H_2_N-Trp-Val-Pro-Ser-Val-Tyr-OH (**4**)	21%

**Scheme 3:**
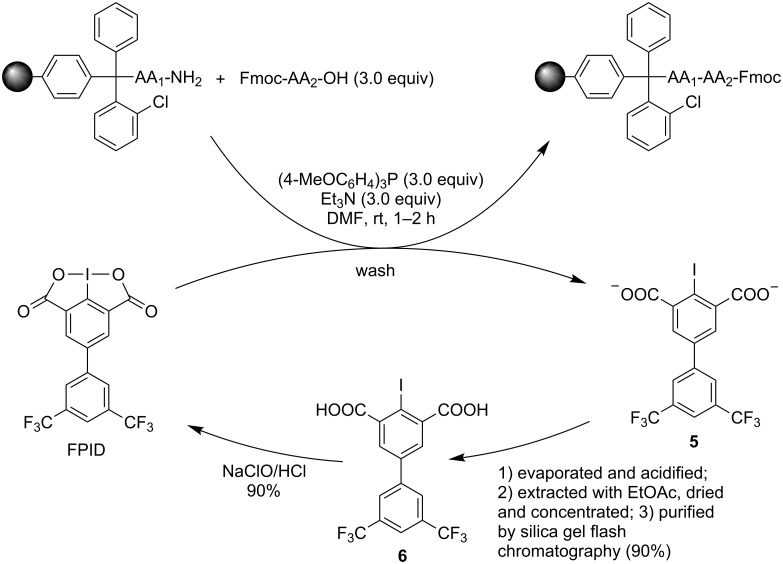
The regeneration of FPID after SPPS.

Similar to the solution-phase peptide synthesis, FPID can be easily regenerated after SPPS (Scheme 3). After completion of peptide elongation, the washing solution of peptide coupling in each cycle was collected and evaporated. Then the mixture was acidified with 3 N HCl and extracted with EtOAc, dried and concentrated in vacuo. The synthetic precursor of FPID **6** could be purified by flash chromatography in order to remove excess Fmoc-protected amino acids during the peptide coupling. Compound **6** was subsequently oxidized with NaOCl/HCl to obtain FPID in 90% yield.

Cyclic peptides, an important kind of peptides, possess several favorable properties such as target selectivity, good binding affinity and low toxicity, which make them attractive candidates in the development of therapeutics [43–44]. Due to the particular significance of cyclic peptides, chemists pay considerable attention to the efficient synthesis of cyclic peptides [45]. In the second part of the investigation of the synthetic utility of the FPID/(4-MeOC_6_H_4_)_3_P system, we tested this system in the cyclic peptide synthesis.

**Figure 2:**
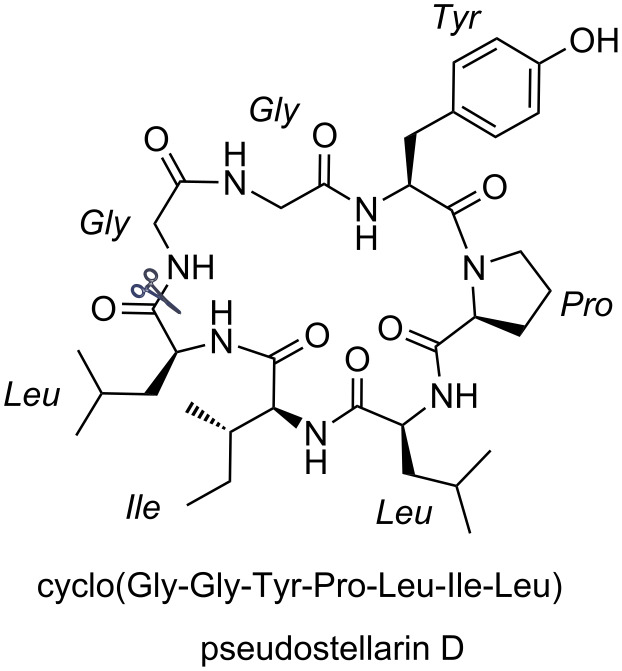
Structure of pseudostellarin D.

**Scheme 4:**
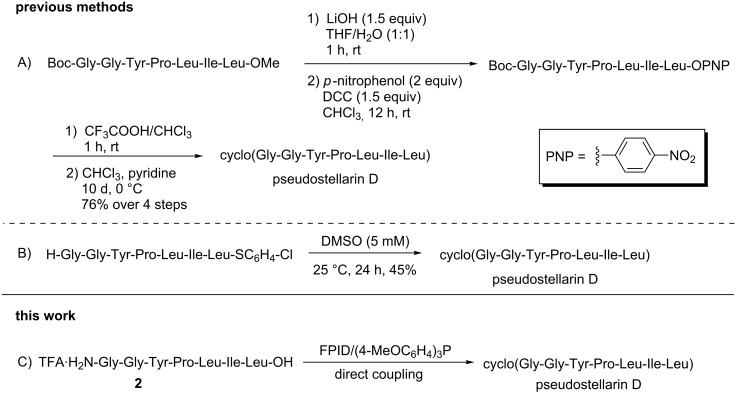
Synthetic strategies of pseudostellarin D.

The roots of *Pseudostellaria heterophylla* are well-known traditional Chinese medicine, which are often used as a lung and spleen tonic. There are more than 10 cyclic peptides isolated from it, for example, pseudostellarin A–H and heterophyllin A–D [37,46–49]. Cyclo(Gly-Gly-Tyr-Pro-Leu-Ile-Leu), a cyclic heptapeptide named pseudostellarin D (Figure 2), is one of these cyclic peptides. Pseudostellarin D was first isolated and identified in 1994 by Itokawa and co-workers [37]. In addition, the authors reported that pseudostellarin D showed potent tyrosinase inhibitory activities. In 1999, Belagali and co-workers further evaluated the antimicrobial, anti-inﬂammatory and anthelmintic activities of pseudostellarin D [38]. As for the synthesis of pseudostellarin D, the existing methods utilized the active ester method to complete the cyclization of the linear peptide precursor with the same amino acid sequence of Gly-Gly-Tyr-Pro-Leu-Ile-Leu as a result of the amide bond between “Gly-Leu” being the cyclization position. The first one was reported by Belagali in 1999, pseudostellarin D was obtained by the cyclization of the linear heptapeptide peptide-PNP ester, which is known as the *p*-nitrophenyl ester method (Scheme 4, method A). The other one was described by Agrigento and co-workers, the cyclization was completed via the *p*-chlorophenyl thioester method with peptide-thioester being the precursor (Scheme 4, method B) [39]. Herein, we realized the synthesis of pseudostellarin D following the same cyclization strategy mentioned above by direct coupling of the precursor **2** without any protecting group utilizing the system of FPID/(4-MeOC_6_H_4_)_3_P (Scheme 4, method C).

**Scheme 5:**
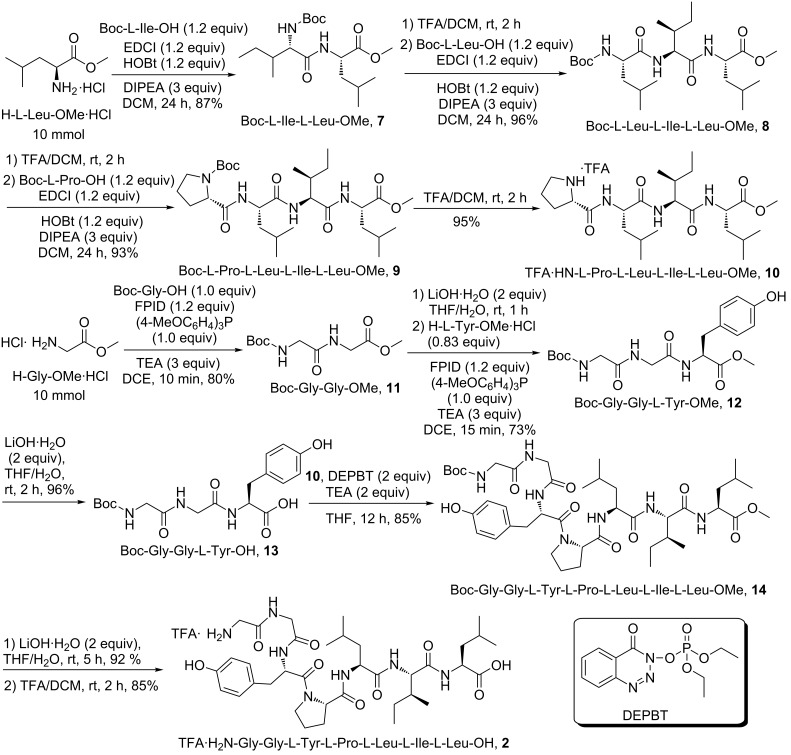
Preparation of the precursor of pseudostellarin D.

We have obtained the precursor **2** through SPPS mediated by FPID/(4-MeOC_6_H_4_)_3_P system (Table 1, entry 2). Firstly, the C-terminal amino acid Leu was immobilized onto 2-Cl-Trt-Cl resin. Then the chain elongation was completed by the deprotection of Fmoc and coupling with Fmoc-protected Ile, Leu, Pro, Tyr, Gly, Gly in turn. The successive deprotection of Fmoc, cleavage from the resin and purification via RP-HPLC yielded the precursor **2** (see Supporting Information File 1). Alternatively, the synthesis of the precursor **2** can be achieved through a convergent [3 + 4] segment condensation strategy in solution phase (Scheme 5). Notably, during the synthesis of tetrapeptide segment **10**, the stepwise manner was adopted in order to avoid racemization. In the synthesis of tripeptide segment **13**, our condensing system, FPID/(4-MeOC_6_H_4_)_3_P was utilized. Besides, the coupling between **10** and **13** to yield heptapeptide segment **14** was carried out using 3-(diethoxyphosphoryloxy)-1,2,3-benzotriazin-4(3*H*)-one (DEPBT) as coupling reagent, which was developed by Ye’s group in order to reduce racemization and side reactions [50]. The precursor **2** was obtained via successive deprotection of the C-terminal and the N-terminal protecting group of **14**. The overall yield of this route is 28%.

With the precursor **2** in hand, we then investigated the reaction conditions of its cyclization. Considering the solubility of **2** and FPID, we chose DMF as the solvent. At the beginning of our investigation, the reaction was carried out under air within 10 h, we tested the influence of the adding sequence of TEA, FPID and (4-MeOC_6_H_4_)_3_P, and the results indicated that adding TEA first and FPID/(4-MeOC_6_H_4_)_3_P 5 minutes later performed better than the inverse sequence (Table 2, entries 1 and 2). When prolonging the reaction time to 24 h, the product was obtained in a yield of 23% and 25% according to different adding sequence of TEA and FPID/(4-MeOC_6_H_4_)_3_P, and the effect of the adding sequence was the same with that mentioned above (Table 2, entries 3 and 4). Then the reaction was run under N_2_ atmosphere instead of air, the cyclization yield increased to 27% (Table 2, entry 5). Increasing the equivalents of FPID and (4-MeOC_6_H_4_)_3_P to 2.0 equiv resulted in 33% NMR yield of the product (Table 2, entry 7). Ye’s group disclosed that adding univalent metal ions such as Na^+^, K^+^ and Cs^+^ to the reaction system for the synthesis of some cyclic pentapeptides and heptapeptide would not only enhance the cyclization yields but also the cyclization rates [51]. Inspired by this finding, we tried to add some metal chlorides into the reaction, such as LiCl, NaCl, KCl, CsCl (Table 2, entries 8–11). The results indicated that adding NaCl, KCl, CsCl could increase the cyclization yield, whereas LiCl decreased the yield. Among the efficient metal chlorides, adding 5 equiv of CsCl to the reaction yielded the product in 44% NMR yield. The isolated yield of pseudostellarin D under the optimized reaction conditions was 32%.

**Table 2:** Optimization of cyclization of linear heptapeptide **2**.^a^

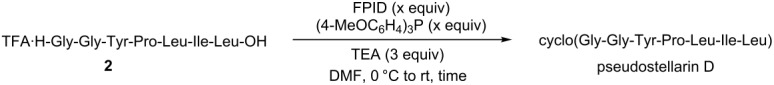					

entry	x equiv	additive	atmosphere	time	yield (%)

1	1.2	–	air	10 h	<18
2^b^	1.2	–	air	10 h	<15
3	1.2	–	air	24 h	<23
4^b^	1.2	–	air	24 h	<20
5	1.2	–	N_2_	24 h	<27
6^c^	2.0	–	N_2_	24 h	n.d.
7	2.0	–	N_2_	24 h	33 (NMR)
8^d^	2.0	CsCl (5 equiv)	N_2_	24 h	44 (NMR)
9^d^	2.0	NaCl (5 equiv)	N_2_	24 h	42 (NMR)
10^d^	2.0	LiCl (5 equiv)	N_2_	24 h	28 (NMR)
11^d^	2.0	KCl (5 equiv)	N_2_	24 h	40 (NMR)

^a^Conditions: performed with **2** (0.05 mmol), TEA (0.15 mmol), DMF (50 mL). Unless otherwise mentioned, the adding sequence of TEA and FPID/(4-MeOC_6_H_4_)_3_P was adding TEA first and FPID/(4-MeOC_6_H_4_)_3_P 5 minutes later. “x equiv” meant the equivalents of FPID and (4-MeOC_6_H_4_)_3_P. The NMR yield was calculated by adding CH_2_ClBr as internal standard substance. ^b^Adding FPID/(4-MeOC_6_H_4_)_3_P first and TEA 5 minutes later. ^c^**2** was dissolved in 2 mL of DMF and added portionwise to the reaction system within 2 h. ^d^Metal chloride was dissolved in 0.33 mL of H_2_O and then added to the reaction.

Consequently, pseudostellarin D can be successfully synthesized in 32% isolated yield (44% NMR yield) under the optimized conditions: FPID (2 equiv), (4-MeOC_6_H_4_)_3_P (2 equiv), TEA (3 equiv), CsCl (5 equiv). The ^1^H NMR and HRMS spectra of pseudostellarin D are in agreement with data from the literature [37–39].

## Conclusion

The system of the hypervalent iodine(III) reagent FPID and (4-MeOC_6_H_4_)_3_P can be applied to the solid-phase peptide synthesis because four bioactive peptides were smoothly obtained including the precursor **2** of cyclic peptide pseudostellarin D. Moreover, we have also successfully synthesized the bioactive cyclic heptapeptide pseudostellarin D using this system. Notably, FPID can be easily regenerated after peptide coupling reaction in SPPS. These results, along with the successful use of the FPID/(4-MeOC_6_H_4_)_3_P system in the solution-phase linear peptide synthesis [29], show its potential in the practical application in peptide synthesis.

## Supporting Information

File 1 Experimental procedures and characterization data of all products, copies of ^1^H, ^13^C, HPLC, HRMS spectra of some compounds.
