# Supplementary material for: Recyclable hypervalent-iodine-mediated solid-phase peptide synthesis and cyclic peptide synthesis
Source: Beilstein J Org Chem. 2018 May 22;14:1112–9. doi: 10.3762/bjoc.14.97 (PMC6009197; doi:10.3762/bjoc.14.97)

Supporting Information

for

Recyclable hypervalent-iodine-mediated solid-phase peptide synthesis and cyclic peptide synthesis

Dan Liu, Ya-Li Guo, Jin Qu and Chi Zhang*

Address: State Key Laboratory of Elemento-Organic Chemistry, Collaborative Innovation Center of Chemical Science and Engineering (Tianjin), College of Chemistry, Nankai University, Tianjin 300071, China

Email: Chi Zhang* - zhangchi@nankai.edu.cn

* Corresponding author

Experimental procedures and characterization data of all products, copies of ^{1}H, ^{13}C, HPLC, HRMS spectra of some compounds

Table of Contents

I. General information......S2

II. Solid-phase peptide synthesis......S3

III. The synthesis of cyclic heptapeptide pseudostellarin D......S7

IV. ^{1}H NMR, ^{13}C NMR, and HPLC spectra of products......S13

I. General information

1. Abbreviations

2-Cl-Trt-Cl resin: 2-Chlorotrityl chloride resin

Fmoc: [(9-Fluorenylmethyl)oxy]carbonyl

DCM: Dichloromethane

DMF: *N*,*N*-Dimethylformamide

DIPEA: *N*,*N*-Diisopropylethylamine

MeOH: Methanol

FPID: (6-(3,5-bis(trifluoromethyl)phenyl)-1*H*,4*H*-2aλ^3-ioda-2,3-dioxacyclopenta[*hi*]indene-1,4-dione)

$(4\text{-}MeOC_6H_4)_3P$: Tris(4-methoxyphenyl)phosphine

TEA: Triethylamine

TFA: Trifluoroacetic acid

Et_2O: Diethyl ether

Boc: *t*-Butoxycarbonyl

EDCI: 1-(3-Dimethylaminopropyl)-3-ethylcarbodiimide hydrochloride

HOBt: 1-Hydroxybenzotriazole

DEPBT: 3-(Diethoxyphosphoryloxy)-1,2,3-benzotrizin-4(3*H*)-one

2. General analytic methods

The ^{1}H NMR spectra were recorded at 400 MHz and ^{13}C NMR spectra were measured at 100 MHz using a Bruker AV400 instrument with $CDCl_3$ or DMSO-d_6 as the solvent. The chemical shifts (δ) were measured in ppm and with the solvents as references (For $CDCl_3$, ^{1}H: δ = 7.26 ppm, ^{13}C: δ = 77.00 ppm; for DMSO-d_6, ^{1}H: δ = 2.50 ppm, ^{13}C: δ = 39.50 ppm). The multiplicities of the signals are described using the following abbreviations: s = singlet, d = doublet, t = triplet, q = quartet, m = multiplet, dd = doublet of doublets, br = broad. High-resolution mass spectral analyses (HRMS) were

performed on a high resolution ESI-FTICR or MALDI-FTICR mass spectrometer (Varian 7.0 T). Reverse phase high performance liquid chromatography (RP-HPLC) analysis was conducted using a Shimadzu LC-10 AD coupled diode array-detector SPD-MA-10A-VP with J&K C18 column. The detector was operated at a wavelength of 214 nm. The RP-HPLC solvent system was acetonitrile with 0.1% TFA added (solvent A) and water with 0.1% TFA added (solvent B). Melting points were recorded on a RY-1 type apparatus. Optical rotations were obtained on a Perkin-Elmer 431 Polarimeter. All solvents were obtained from commercial sources and were purified according to standard procedures. Petroleum ether (PE), where used, had the boiling point range 60–90 °C.

II. Solid-phase peptide synthesis

1. General Procedure for Fmoc-SPPS mediated by FPID

All Fmoc-protected amino acids and 2-chlorotrityl chloride resin (2-Cl-Trt-Cl resin, 0.98 mmol/g) were purchased and used without further purification. All peptides were synthesized using 200 mg resin in solid-phase peptide synthesis vessel. The synthetic route included immobilized the C-terminal amino acid onto resin, deprotection, coupling and cleavage from resin.

Immobilized the C-terminal amino acid onto resin:

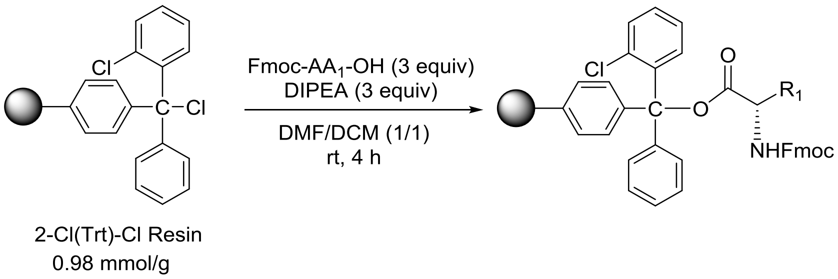

Procedure: 2-Cl-Trt-Cl resin (0.196 mmol, 200 mg) and C-terminal Fmoc-amino acid (Fmoc-AA_1-OH, 0.6 mmol) were added to a 5 mL round-bottom flask, then DCM and DMF (2 mL, v:v = 1:1) were added and the suspension was stirred at room temperature. DIPEA (0.1 mL) was then added and the reaction was stirred at room temperature for 4 h. The reaction was filtered and washed with MeOH (3 × 5 mL) and DCM (3 × 5 mL). The resin was used in next step without further purification. For the reaction sites that failed to immobilize the C-terminal amino acid, blocking these reaction sites is necessary.

Blocked the reaction sites:

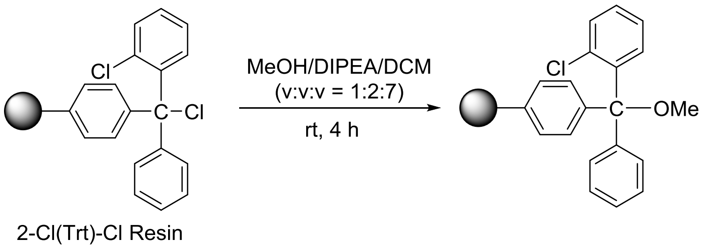

Procedure: To the resin from last step in a 5 mL round-bottom flask, was added MeOH/DIPEA/DCM (2 mL, v:v:v = 1:2:7) and the reaction was stirred at room temperature for 4 h. The reaction was filtered and washed with MeOH (3 × 5 mL) and DCM (3 × 5 mL). The resin was used in next step without further purification.

Deprotection of Fmoc:

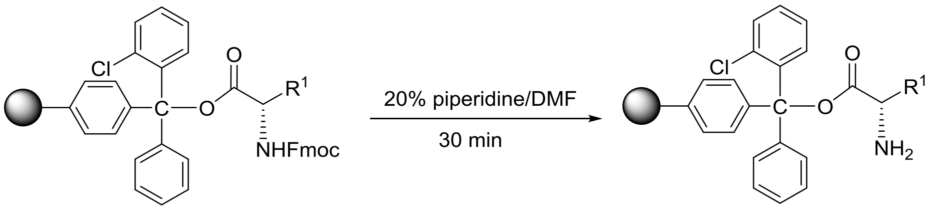

Procedure: To the resin from last step in solid-phase peptide synthesis vessel was added 20% piperidine/DMF (2mL) and the reaction was agitated with air bubbling from the bottom of the reaction vessel by using air-pump. In order to make sure the completely deprotection of Fmoc, the reaction last 30 minutes. After 30 min, the reaction was filtered and washed with MeOH (3 × 5 mL) and DCM (3 × 5 mL). The resin was used in next step without further purification.

Coupling:

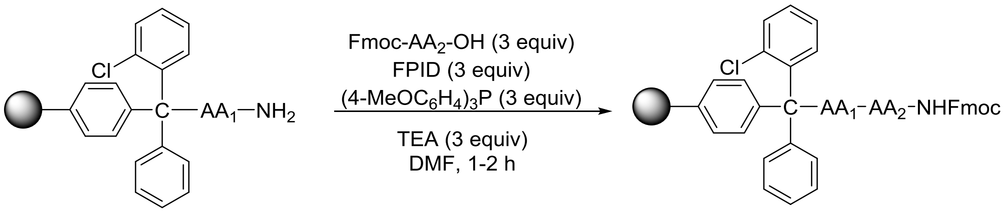

Note: Fmoc-Ser-OH, Fmoc-Thr-OH, Fmoc-Tyr-OH, Fmoc-Trp-OH were directly used without any protecting group on OH or NH. During the synthesized of **3**, Fmoc-Lys(Boc)-OH was used.

Procedure: To the resin from last step in solid phase peptide synthesis vessel, was added Fmoc-AA_2-OH (0.6 mmol) and DMF (2 mL), and then the reaction was agitated with air bubbling from the bottom of the reaction vessel by using air-pump. TEA (0.6 mmol, 84 μL) was then added. FPID (305 mg, 0.6 mmol) and $(4\text{-MeOC}_6H_4)_3P$ (116 mg, 0.6 mmol) was added 5 min later. After 1-2 h, the reaction was

filtered and washed with MeOH (3 × 5 mL) and DCM (3 × 5 mL). The resin was used in next step without further purification. The peptide chain elongation was completed by repeating deprotection of Fmoc and peptide coupling reaction.

Cleavage:

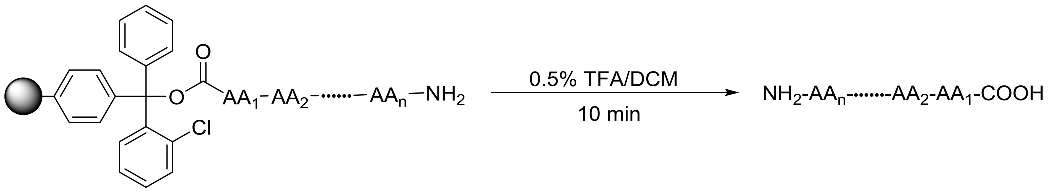

Procedure: To the resin from last step in solid phase peptide synthesis vessel, was added 0.5% TFA/DCM (2 mL). The reaction was agitated for 10 min with air bubbling from the bottom of the reaction vessel by using air-pump. Then the reaction was filtered and washed with MeOH (3 × 5 mL) and DCM (3 × 5 mL). The filtrate was collected and evaporated and the crude peptide was precipitated from Et_2O, dried in vacuo.

2. Purification of peptides

The crude peptides were purified via RP-HPLC. Analytical HPLC was performed using J&K C18 column (5 µm, 4.6 mm × 250 mm) at a flow rate of 0.8 mL·min^{-1}. Preparative HPLC was performed using Shimpack Prep-ODS C18 column (15 µm, 20 mm × 250 mm) at a flow rate of 8 mL·min^{-1}.

3. Regeneration of FPID

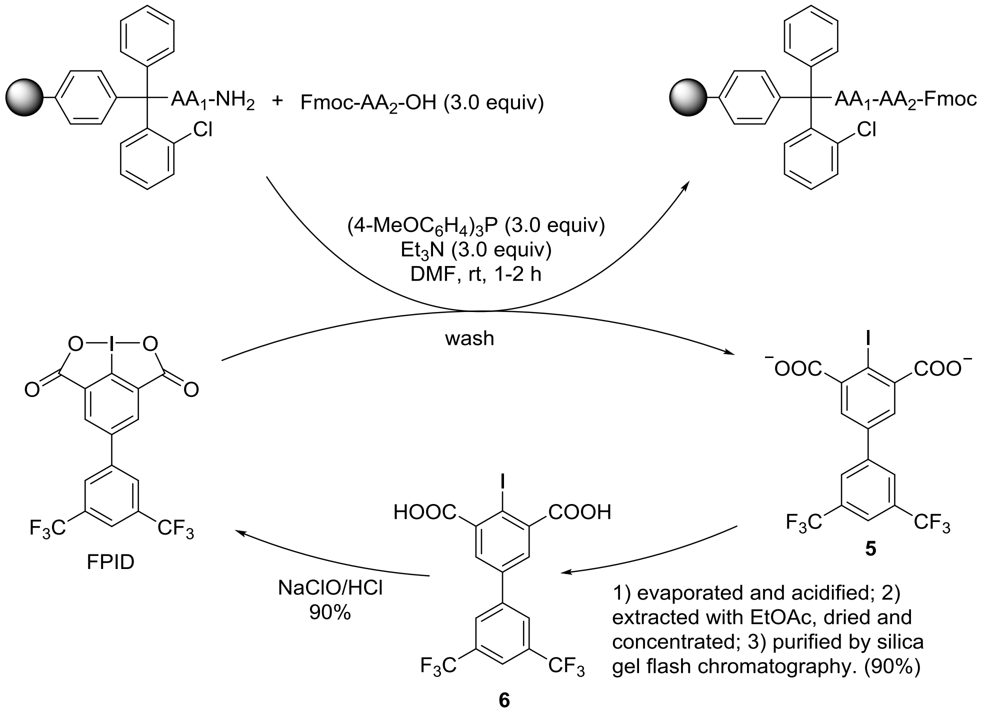

Recovery of the precursor of FPID 6: After completion of peptide elongation, the washing solution of peptide coupling in each cycle was collected and evaporated. Then the mixture was acidified with 3 N HCl and extracted with EtOAc, dried and concentrated in vacuo. In order to remove excess Fmoc-amino acids during the peptide coupling, the precursor of FPID **6** could be purified by silica gel flash chromatography in 90% yield.

Oxidation of 6: The compound **6** (504 mg, 1 mmol) was dispersed in conc. HCl (10 mL), and aq. 10% NaOCl soln. (20 mL) was added dropwise over 30 min to this vigorously stirred suspension at room temperature. Stirring was continued for a further 4 h when the addition of NaOCl was complete. The precipitated colorless solid was collected by filtration, washed with water, and dried at room temperature to obtain FPID (450 mg, 90%). Colorless solid; ^{1}H NMR (400 MHz, DMSO-d_6): δ = 8.77 (s, 2H), 8.61 (s, 2H), 8.21 (s, 1H).

4. Characterization of Products

H_2N-Tyr-Gly-Gly-Phe-Leu-OH (1): The peptide was obtained 47 mg colorless solid in the yield of 42%. R_t = 48.640 min. HRMS (MALDI): *m/z* calcd. for $C_{28}H_{37}N_5O_7$ $[M+Na]^+$: 578.2585, found: 578.2588.

H_2N-Gly-Gly-Tyr-Pro-Leu-Ile-Leu-OH (2): The peptide was obtained 78 mg colorless solid in the yield of 53%. R_t = 38.102 min. HRMS (MALDI): *m/z* calcd. for $C_{36}H_{57}N_7O_9$ $[M+Na]^+$: 754.4110, found: 754.4115.

H_2N-Lys-Leu-Pro-Ala-Gly-Thr-Leu-Phe-OH (3): The peptide was obtained 50 mg colorless solid in the yield of 30%. R_t = 45.807 min. HRMS (MALDI): *m/z* calcd. for $C_{41}H_{67}N_9O_{10}$ $[M+Na]^+$: 868.4903, found: 868.4907.

H_2N-Trp-Val-Pro-Ser-Val-Tyr-OH (4): The peptide was obtained 32 mg colorless solid in the yield of 21%. R_t = 73.243 min. HRMS (MALDI): *m/z* calcd. for $C_{38}H_{51}N_7O_9$ $[M+Na]^+$: 772.3640, found: 772.3645.

III. The synthesis of cyclic heptapeptide pseudostellarin D

1. Preparation of the linear precusor heptapeptide

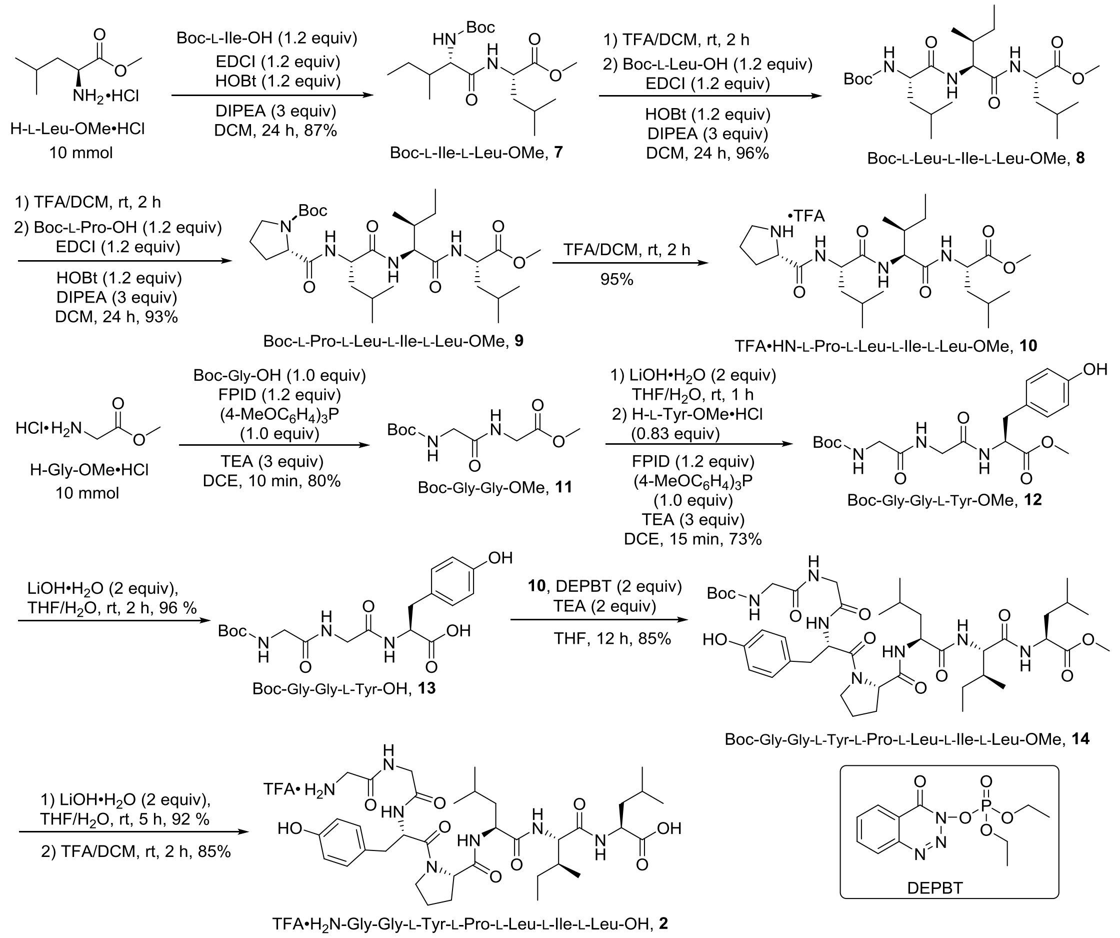

General procedure for peptide coupling mediated by EDCI/HOBt: To a solution of Boc-AA_1-OH (1.2 equiv) in DCM (0.34 M), was added EDCI (1.2 equiv) and HOBt (1.2 equiv). The resulting mixture was stirred at 0 °C for 30 min. H_2N-AA_2-OMe·HCl or H_2N-AA_3-AA_4-OMe·TFA (1.0 equiv) in DCM and DIPEA were then added. The reaction was stirred at rt for 24 h. The reaction was diluted with EtOAc, washed with 10% citric acid solution (3 × 20 mL), water (3 × 20 mL), saturated $NaHCO_3$ (2 × 20 mL), water (2 × 20 mL), brine (1 × 20 mL), and dried over anhydrous $MgSO_4$, filtered and evaporated. The residue was purified by silica gel flash chromatography.

General procedure for peptide coupling mediated by FPID/(4-MeOC_6H_4)$_3$P: To a solution of Boc-AA_1-OH (1.0 equiv), H_2N-AA_2-OMe·HCl or H_2N-AA_3-AA_4-OMe·TFA (1.0 equiv) in DCE (0.03 M), was added Et_3N (3.0 equiv). The solution was stirred at room temperature for 5 min. Then, FPID (1.2 equiv) and (4-MeOC_6H_4)$_3$P (1.0 equiv) were added in sequence. The resulting mixture was stirred at room temperature and monitored by TLC. The reaction was quenched with saturated aq. sodium bicarbonate solution (10 mL) and extracted with EtOAc (50 mL × 3). The combined organic layer was washed with brine, dried over anhydrous $MgSO_4$, filtered and evaporated. The residue was purified by silica gel flash chromatography.

General procedure for peptide coupling mediated by DEPBT: To a solution of Boc-AA_1-OH (1.0 equiv), H_2N-AA_2-OMe·HCl or H_2N-AA_3-AA_4-OMe·TFA (1.0 equiv) and DEPBT (2.0 equiv) in THF (0.33 M), was added TEA (2.0 equiv). The reaction was stirred at rt for 12 h. Saturated NaCl solution (10 mL) was added. The peptide was extracted with EtOAc (3×20 mL). The combined organic layer was washed with 1N HCl (1 × 20 mL), water (1 × 20 mL), 5% Na_2CO_3 (1 × 20 mL), brine (1 × 20 mL) and dried over anhydrous $MgSO_4$, filtered and evaporated. The residue was purified by silica gel flash chromatography.

General procedure for Boc deprotection: To a solution of Boc-protected peptide chain (1.0 equiv) in DCM (0.1 M), was added TFA (3 mL/mmol) dropwise at 0 °C. After 30 min, the mixture was allowed to warm to room temperature and stirred for 1.5 h. After the reaction was finished, the solvent was removed under reduced pressure and the addition of ether to the residue led to the precipitation. The product was used into next step without further purification.

General procedure for ester deprotection: To a solution of Boc-AA_5-AA_6-OMe (1.0 equiv) in THF/H_2O (10 mL/mmol, v:v = 1:1), was added LiOH·H_2O (2.0 equiv). The reaction was stirred at rt. After the reaction was finished, the reaction mixture was concentrated and acidified with 2 N HCl (aq) solution. The peptide was extracted with EtOAc (3 × 20 mL). The combined organic layer was

washed with brine (1 × 20 mL), dried over anhydrous $MgSO_4$, filtered and evaporated. The product was used into next step without further purification.

2. Synthesis of pseudostellarin D

General procedure for cyclization (non-metal ion): To a solution of TFA·H_2N-Gly-Gly-L-Tyr-L-Pro-L-Leu-L-Ile-L-Leu-OH (**2**, 42.3 mg, 0.05 mmol) in DMF (50 mL) under N_2 atmosphere at 0 °C, was added TEA (21 μL, 0.15 mmol). The resulting mixture was stirred for 5 min and then FPID (50.2 mg, 0.1 mmol) and (4-$MeOC_6H_4)_3P$ (35.2 mg, 0.1 mmol) were added in turn. The reaction was stirred at 0 °C for 30 min and then warmed to room temperature for 24 h. After the reaction was finished, the solvent was removed in vacuo. The crude product was purified by silica gel flash chromatography to obtain Pseudostellarin D [*cyclo*(Gly-Gly-Tyr-Pro-Leu-Ile-Leu)].

General procedure for cyclization (with metal ion): To a solution of TFA·H_2N-Gly-Gly-L-Tyr-L-Pro-L-Leu-L-Ile-L-Leu-OH (**2**, 42.3 mg, 0.05 mmol) in DMF (50 mL) under N_2 atmosphere at 0 °C, was added TEA (21 μL, 0.15 mmol). The resulting mixture was stirred for 5 min and then FPID (50.2 mg, 0.1 mmol) and (4-$MeOC_6H_4)_3P$ (35.2 mg, 0.1 mmol) were added in turn. MCl (0.25 mmol) in H_2O (0.33 mL) was added to the reaction. The reaction was stirred at 0 °C for 30 min and then warmed to room temperature for 24 h. After the reaction was finished, the solvent was removed in vacuo. The crude product was purified by silica gel flash chromatography to obtain Pseudostellarin D [*cyclo*(Gly-Gly-Tyr-Pro-Leu-Ile-Leu)].

3. Characterization of products

***N-tert*-Butoxycarbonyl-L-isoleucyl-L-leucine methyl ester (7)**

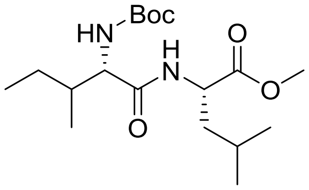

Boc-L-Ile-L-Leu-OMe (7): The reaction afforded 3.11 g of compound **7** in 87% yield as colorless solid; ^{1}H NMR (400 MHz, $CDCl_3$): δ = 6.30 (d, J = 6.8 Hz, 1H), 5.07 (d, J = 7.6 Hz, 1H), 4.63-4.58 (m, 1H), 3.93-3.90 (m, 1H), 3.71 (s, 3H), 1.84 (s, 1H), 1.66-1.42 (m, 13H), 1.19-1.08 (m, 1H), 0.93-0.87 (m, 12H); ^{13}C NMR (100 MHz, $CDCl_3$): δ = 173.0, 171.4, 155.7, 79.8, 59.2, 52.2, 50.6, 41.4, 37.1, 28.2, 24.7, 22.8, 21.8, 15.4, 11.3; HRMS (ESI): m/z calcd. for $C_{18}H_{34}N_2O_5$ $[M+H]^+$: 359.2540, found 359.2541.

***N-tert*-Butoxycarbonyl-L-leucyl-L-isoleucyl-L-leucine methyl ester (8)**

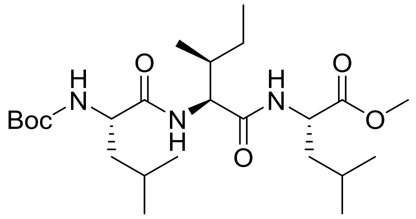

Boc-L-Leu-L-Ile-L-Leu-OMe (8): The reaction afforded 3.94 g of compound **8** in 96% yield as colorless solid; ^{1}H NMR (400 MHz, $CDCl_3$): δ = 6.64 (d, J = 8.4 Hz, 1H), 6.40 (d, J = 6.4 Hz, 1H), 4.92 (d, J = 6.8 Hz, 1H), 4.61-4.56 (m, 1H), 4.27 (t, J = 7.9 Hz, 1H), 4.08 (d, J = 6.4 Hz, 1H), 3.72 (s, 3H), 1.91 (s, 1H), 1.70-1.43 (m, 16H), 1.19-1.08 (m, 1H), 0.94-0.87 (m, 18H); ^{13}C NMR (100 MHz, $CDCl_3$): δ = 173.1, 172.6, 171.1, 155.6, 79.7, 57.6, 53.1, 52.1, 50.6, 41.0, 40.9, 36.9, 28.2, 24.7, 24.7, 22.8, 22.7, 22.1, 21.8, 15.2, 11.2; HRMS (ESI): *m/z* calcd. for $C_{24}H_{45}N_3O_6$ $[M+H]^+$:472.3381, found: 472.3379.

***N-tert*-Butoxycarbonyl- L-prolyl-L-leucyl-L-isoleucyl-L-leucine methyl ester (9)**

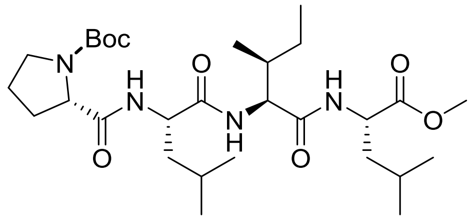

Boc- L-Pro-L-Leu-L-Ile-L-Leu-OMe (9): The reaction afforded 4.40 g of compound **9** in 93% yield as colorless solid; $[\alpha]_D$: + 12.5° (c = 1.0, CH_3OH); ^{1}H NMR (400 MHz, $CDCl_3$): δ = 7.08 (br s, 1H), 6.79 (br s, 1H), 6.58 (br s, 1H), 4.57 (s, 1H), 4.32-4.24 (m, 3H), 3.71 (s, 3H), 3.41 (s, 2H), 2.23-1.46 (m, 21H), 1.17 (s, 1H), 0.94-0.86 (m, 18H); ^{13}C NMR (100 MHz, $CDCl_3$): δ = 172.9, 172.3, 171.9, 171.1, 155.4, 154.2, 80.2, 79.8, 59.9, 57.5, 51.9, 51.4, 50.6, 47.0, 46.7, 41.8, 40.7, 37.1, 36.6, 31.1, 29.5, 28.5, 28.1, 24.6, 23.4, 22.7, 22.6, 21.6, 15.0, 11.1; HRMS (ESI): *m/z* calcd. for $C_{29}H_{52}N_4O_7$ $[M+H]^+$: 569.3909, found: 569.3919.

***N-tert*-Butoxycarbonylglycyl-glycine methyl ester (11)**

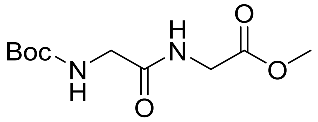

Boc-Gly-Gly-OMe (11): The reaction afforded 1.96 g of compound **11** in 80% yield as yellowish solid; ^{1}H NMR (400 MHz, $CDCl_3$): δ = 6.59 (br s, 1H), 5.12 (br s, 1H), 4.07 (d, J = 5.6 Hz, 2H), 3.85

(d, *J* = 6.0 Hz, 2H), 3.76 (s, 3H), 1.46 (s, 9H); ^{13}C NMR (100 MHz, $CDCl_3$): δ = 170.2, 169.8, 156.1, 80.4, 52.4, 44.2, 41.0, 28.2.

***N-tert*-Butoxycarbonylglycyl-glycyl- L-tyrosine methyl ester (12)**

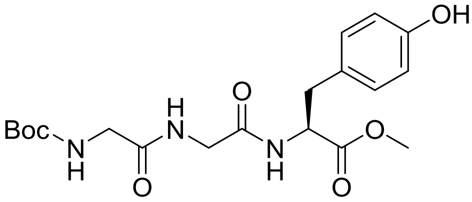

Boc-Gly-Gly-L-Tyr-OMe (12): The reaction afforded 2.40 g of compound **12** in 73% yield as colorless solid; $[\alpha]_D$: + 7.8° (c = 1.0, CH_3OH); ^{1}H NMR (400 MHz, $CDCl_3$): δ = 7.53 (br s, 1H), 7.16 (br s, 1H), 7.09 (d, *J* = 8.4 Hz, 1H), 6.92 (d, *J* = 8.4 Hz, 2H), 6.69 (d, *J* = 8.4 Hz, 2H), 5.55 (t, *J* = 5.8 Hz, 1H), 4.82-4.77 (m, 1H), 3.81-3.73 (m, 6H), 3.11-3.06 (m, 1H), 2.94-2.88 (m, 1H), 1.44 (s, 9H). ^{13}C NMR (100 MHz, $CDCl_3$): δ = 172.1, 170.6, 169.2, 156.5, 155.7, 130.3, 126.9, 115.6, 80.5, 53.6, 52.4, 43.9, 42.6, 36.8, 28.3; HRMS (ESI): *m*/*z* calcd. for $C_{19}H_{27}N_3O_7$ $[M+H]^+$: 410.1922, found: 410.1923.

***N-tert*-Butoxycarbonylglycyl-glycyl--L-tyrosyl-L-prolyl-L-leucyl-L-isoleucyl-L-leucine methyl ester (14)**

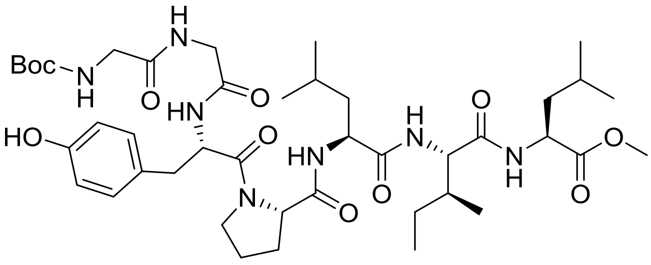

Boc-Gly-Gly-L-Tyr-L-Pro-L-Leu-L-Ile-L-Leu-OMe (14): The reaction afforded 360 mg of compound **14** in 85% yield as colorless solid; $[\alpha]_D$: –61.5° (c = 1.0, CH_3OH); ^{1}H NMR (400 MHz, $CDCl_3$): *δ* = 7.66-7.49 (m, 4H), 7.15-7.11 (m, 2H), 6.98 (d, *J* = 7.6 Hz, 2H), 6.73 (d, *J* = 8.4 Hz, 2H), 5.61 (s, 1H), 4.61 (s, 1H), 4.43-4.42 (m, 1H), 4.25-4.13 (m, 4H), 3.92-3.57 (m, 7H), 2.94-2.81 (m, 3H), 2.03-1.45 (m, 21H), 1.16-1.13 (m, 1H), 0.92-0.86 (m, 18H); ^{13}C NMR (100 MHz, $CDCl_3$): δ = 173.1, 172.5, 171.9, 171.8, 171.2, 170.2, 169.9, 156.3, 155.9, 130.2, 126.6, 115.4, 80.0, 60.6, 57.8, 54.0, 53.0, 52.1, 51.2, 46.9, 44.1, 42.3, 40.2, 39.5, 36.8, 35.7, 28.2, 24.8, 24.6, 24.5, 22.9, 22.6, 21.6, 21.5, 15.2, 10.7; HRMS (ESI): *m*/*z* calcd. for $C_{42}H_{67}N_7O_{11}$ $[M+H]^+$: 846.4971, found: 846.4965.

Pseudostellarin D [*cyclo*(Gly-Gly-Tyr-Pro-Leu-Ile-Leu)]

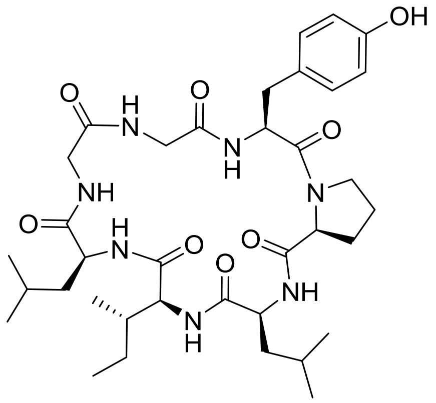

***Cyclo*(Gly-Gly-Tyr-Pro-Leu-Ile-Leu):** The reaction afforded 11.5 mg of Pseudostellarin D in 32% yield as colorless solid in the presence of 5.0 equiv CsCl; m.p. 176-178 °C (lit. 177-179 °C), $[\alpha]_D$: –66.3° (lit. –64.8°)(c = 0.01, CH_3OH), ^1H NMR (400 MHz, DMSO-d_6): δ = 9.40-9.20 (m, 1H), 8.73 (s, 1H), 8.59- 8.46 (m, 1H), 8.30-8.17 (m, 1H), 7.97 (m, 1H), 7.71 (d, J = 6.3 Hz, 1H), 7.49-7.31 (m, 1H), 7.10-6.94 (m, 2H), 6.70-6.65 (m, 2H), 4.70 (m, 1H), 4.45 (s, 1H), 4.28 (s, 1H), 4.16-4.08 (m, 2H), 3.95-3.85 (m, 2H), 3.79-3.67 (m, 2H), 3.58-3.44 (m, 2H), 3.17-2.98 (m, 1H), 2.86-2.67 (m, 1H), 2.18 (s, 1H), 2.00-1.40 (m, 12H), 1.22-1.07 (m, 2H), 0.88-0.62 (m, 18H); HRMS (MALDI): *m*/*z* calcd. for $C_{36}H_{55}N_7O_8$ $[M+Na]^+$: 736.4004, found: 736.4008.

IV. ^{1}H NMR, ^{13}C NMR, and HPLC spectra of products

H_2N-Tyr-Gly-Gly-Phe-Leu-OH (1)

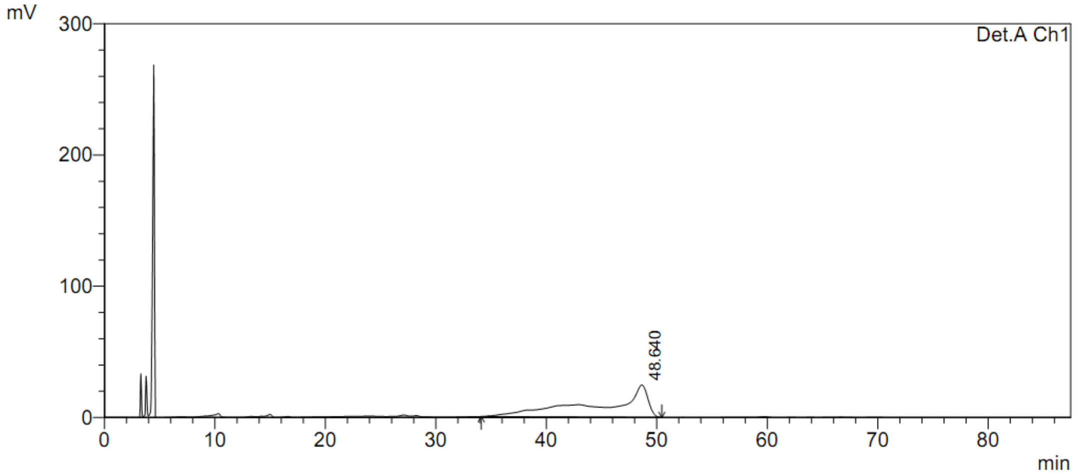

1 Det.A Ch1/214nm

PeakTable

Detector A Ch1 214nm

Peak#	Ret. Time	Area	Height	Area %	Height %
1	48.640	7090870	24774	100.000	100.000
Total		7090870	24774	100.000	100.000

RP-HPLC Condition: 80% B, R_t = 48.640 min

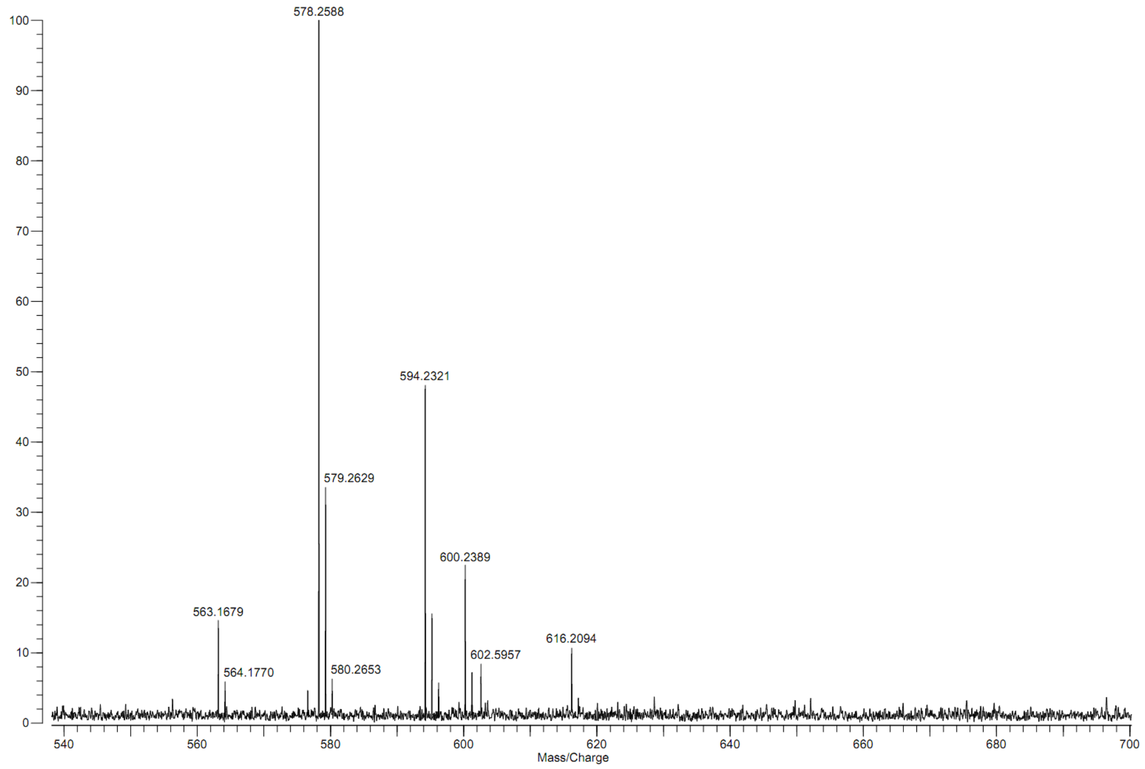

H_2N-Gly-Gly-Tyr-Pro-Leu-Ile-Leu-OH (2)

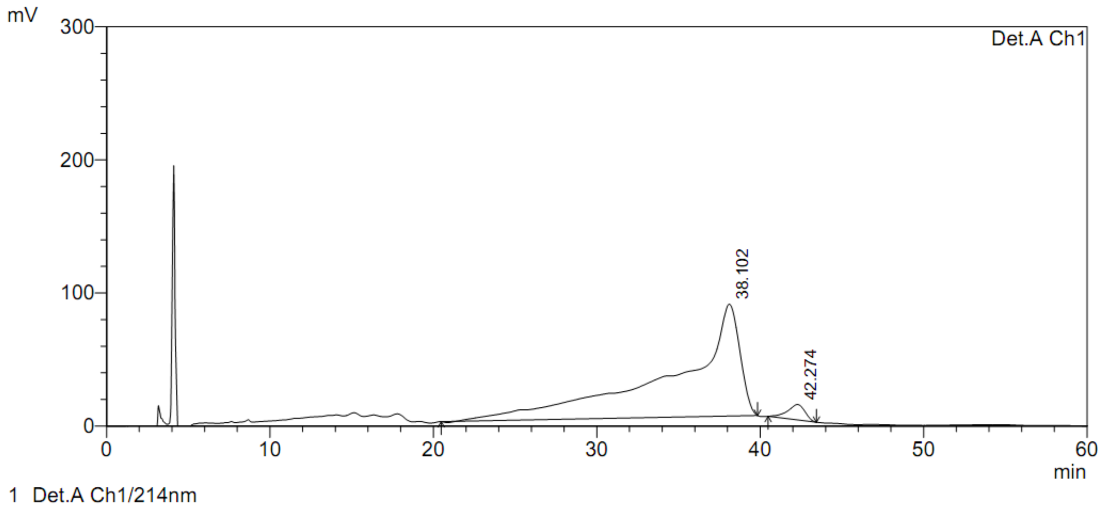

PeakTable

Detector A Ch1 214nm

Peak#	Ret. Time	Area	Height	Area %	Height %
1	38.102	23485208	84046	96.466	87.935
2	42.274	860455	11531	3.534	12.065
Total		24345663	95577	100.000	100.000

RP-HPLC Condition: 75% B, R_t = 38.102 min

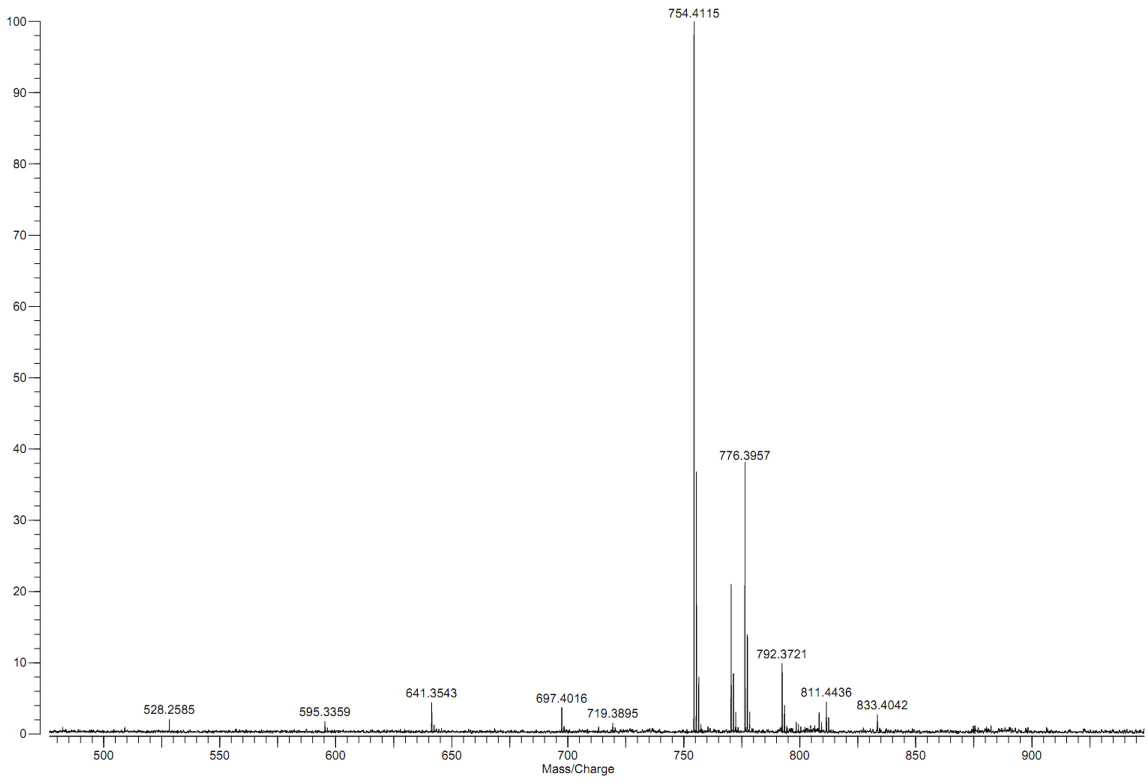

H_2N-Lys-Leu-Pro-Ala-Gly-Thr-Leu-Phe-OH (3)

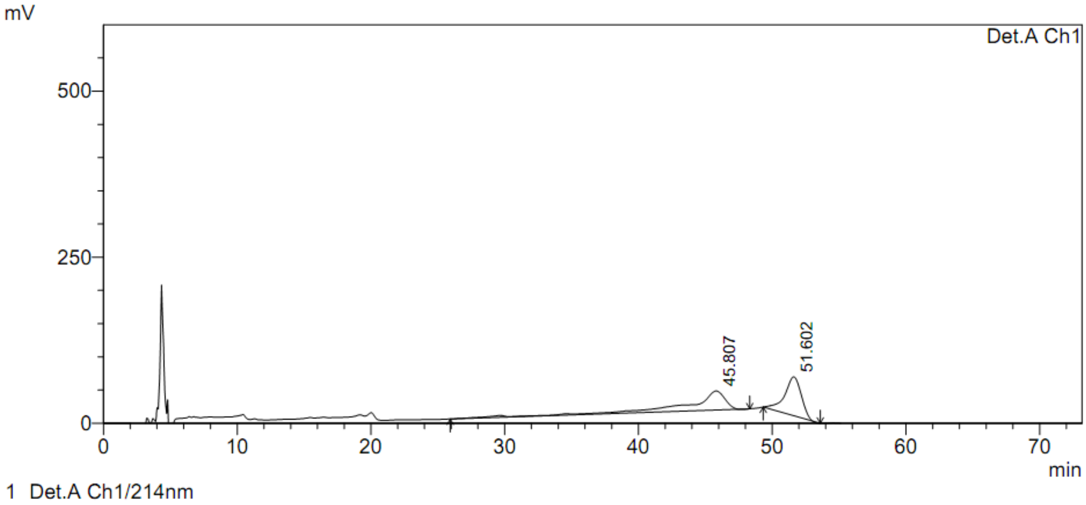

PeakTable

Detector A Ch1 214nm

Peak#	Ret. Time	Area	Height	Area %	Height %
1	45.807	5791856	28648	53.445	32.883
2	51.602	5045199	58473	46.555	67.117
Total		10837055	87121	100.000	100.000

RP-HPLC Condition: 80% B, R_t = 45.807 min

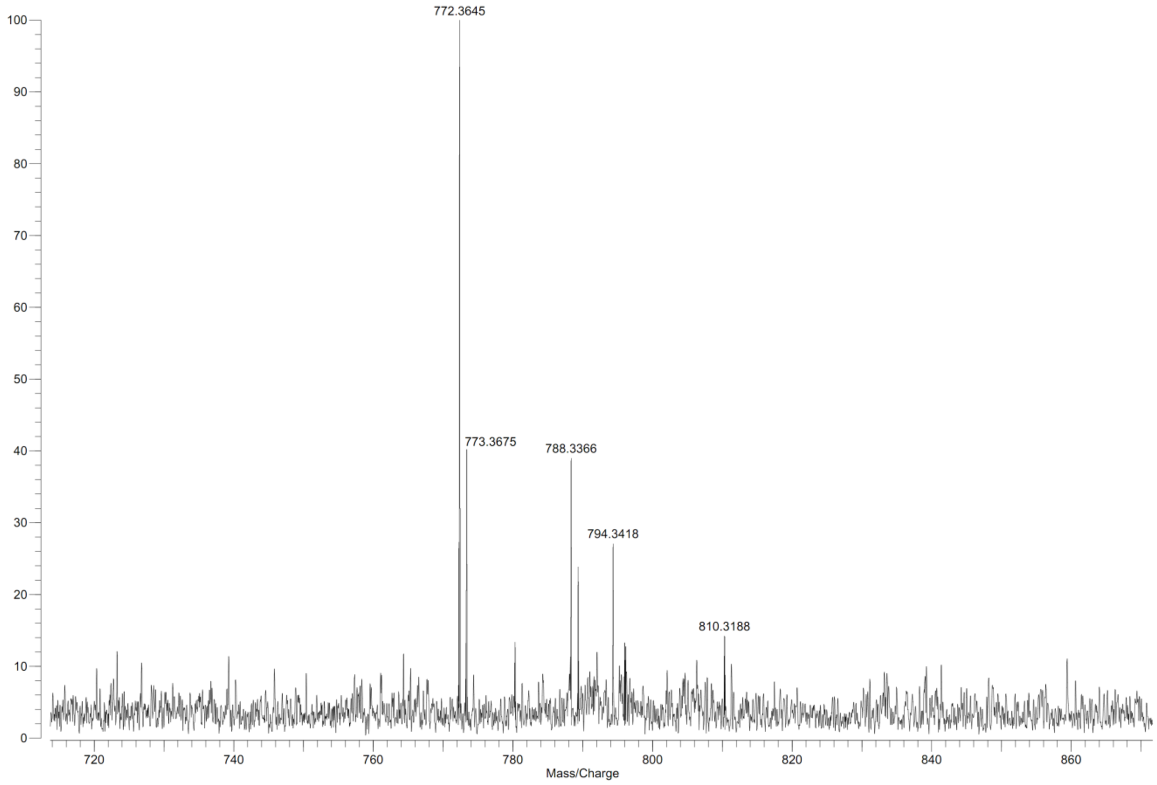

H_2N-Trp-Val-Pro-Ser-Val-Tyr-OH (4)

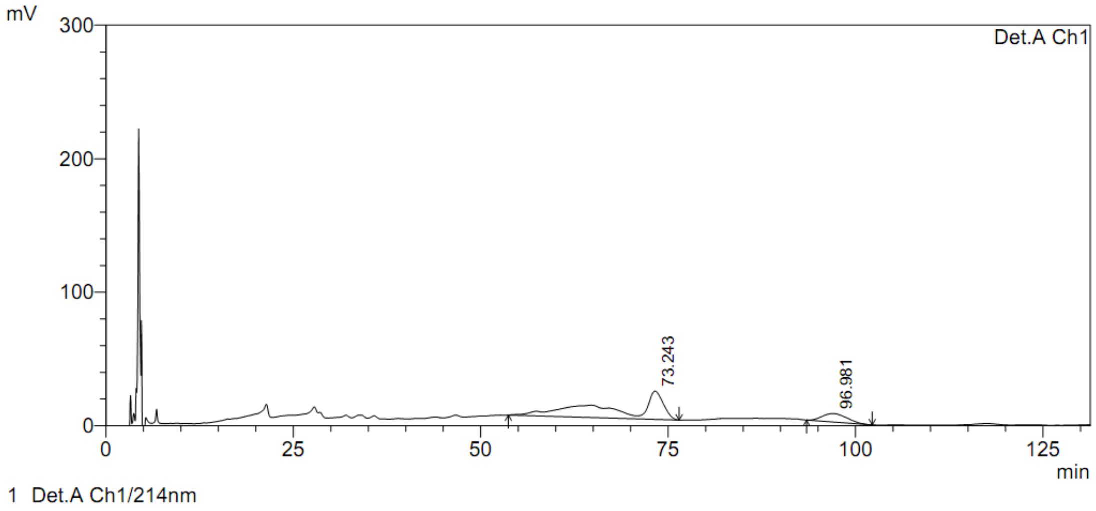

PeakTable

Detector A Ch1 214nm

Peak#	Ret. Time	Area	Height	Area %	Height %
1	73.243	8164934	21180	84.875	76.918
2	96.981	1454968	6356	15.125	23.082
Total		9619902	27535	100.000	100.000

RP-HPLC Condition: 80% B, R_t = 73.243 min

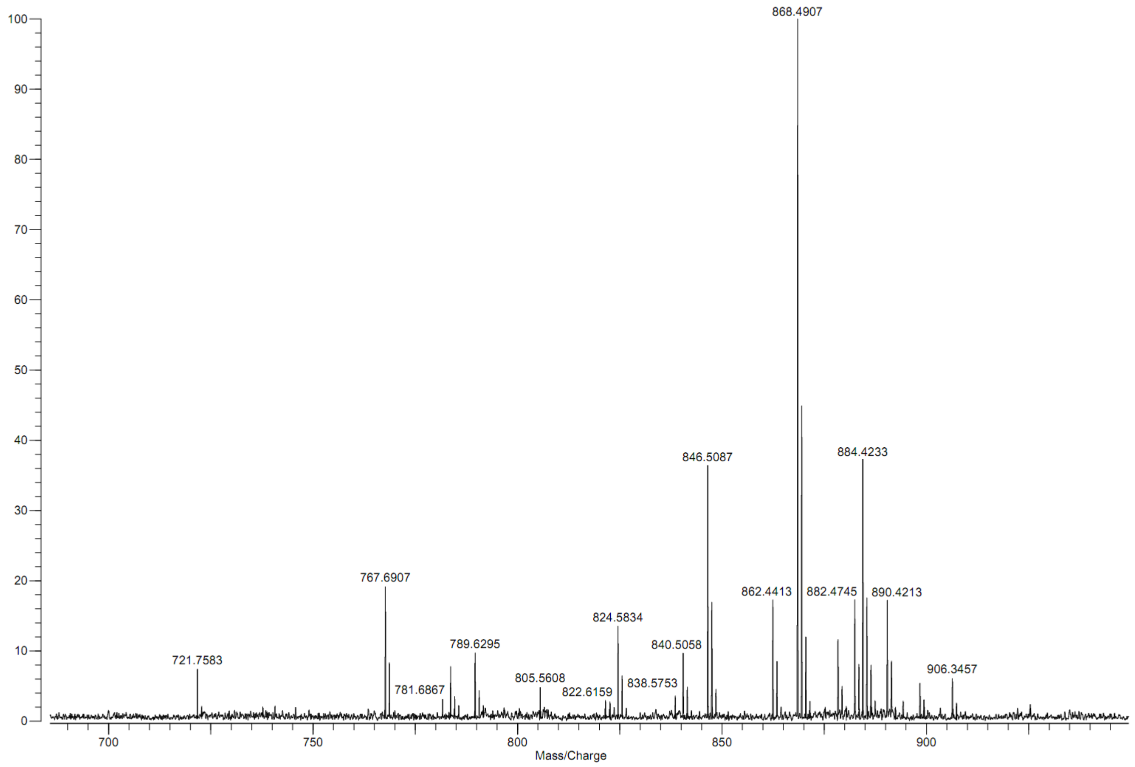

FPID

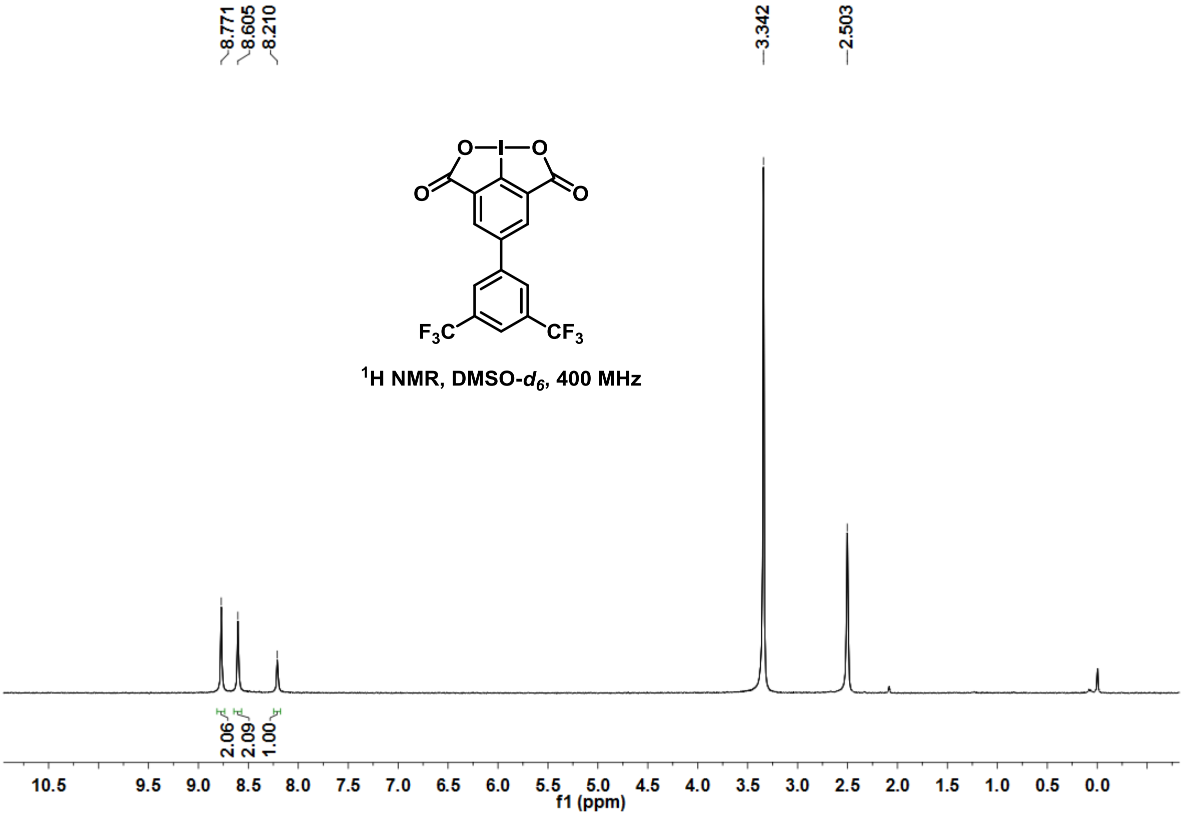
8.771
8.605
8.210
3.342
2.503
^{1}H NMR, DMSO-d_6, 400 MHz
2.06
2.09
1.00
f1 (ppm)

Boc-L-Ile-L-Leu-OMe (7)

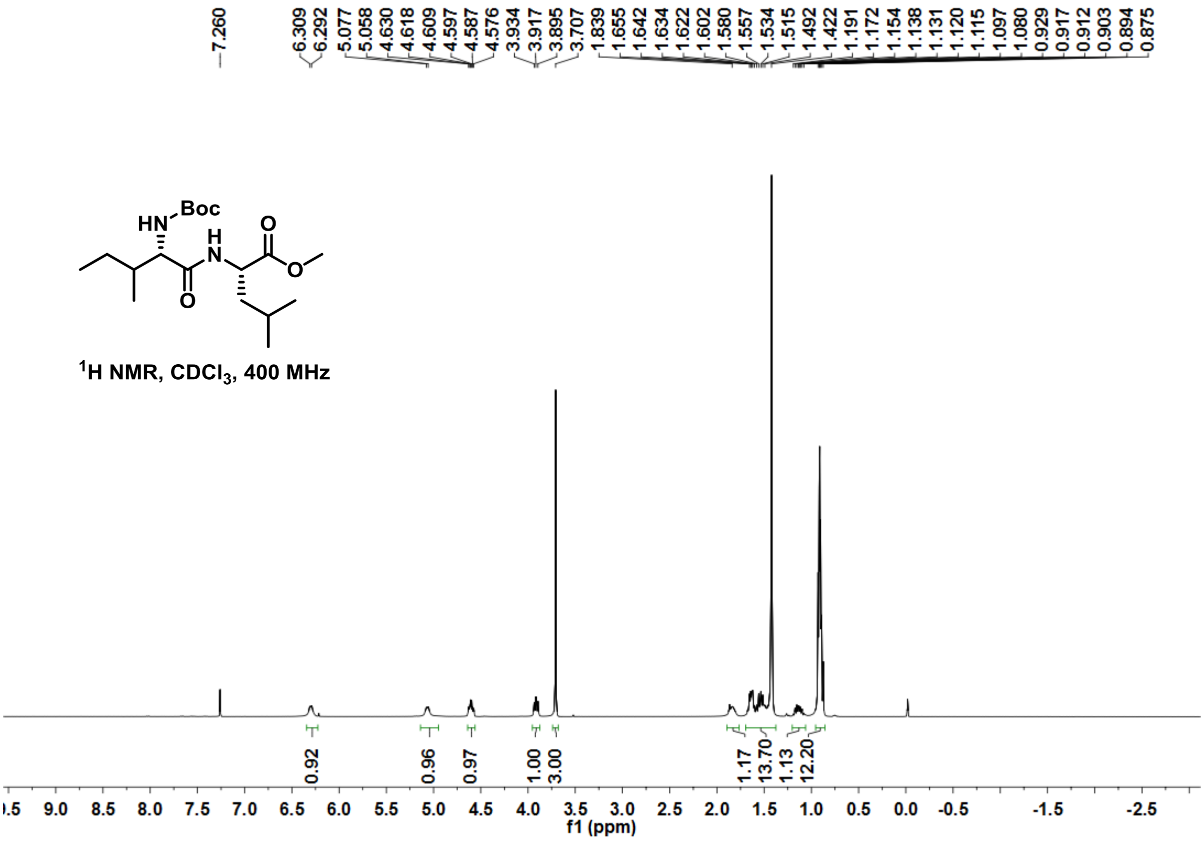

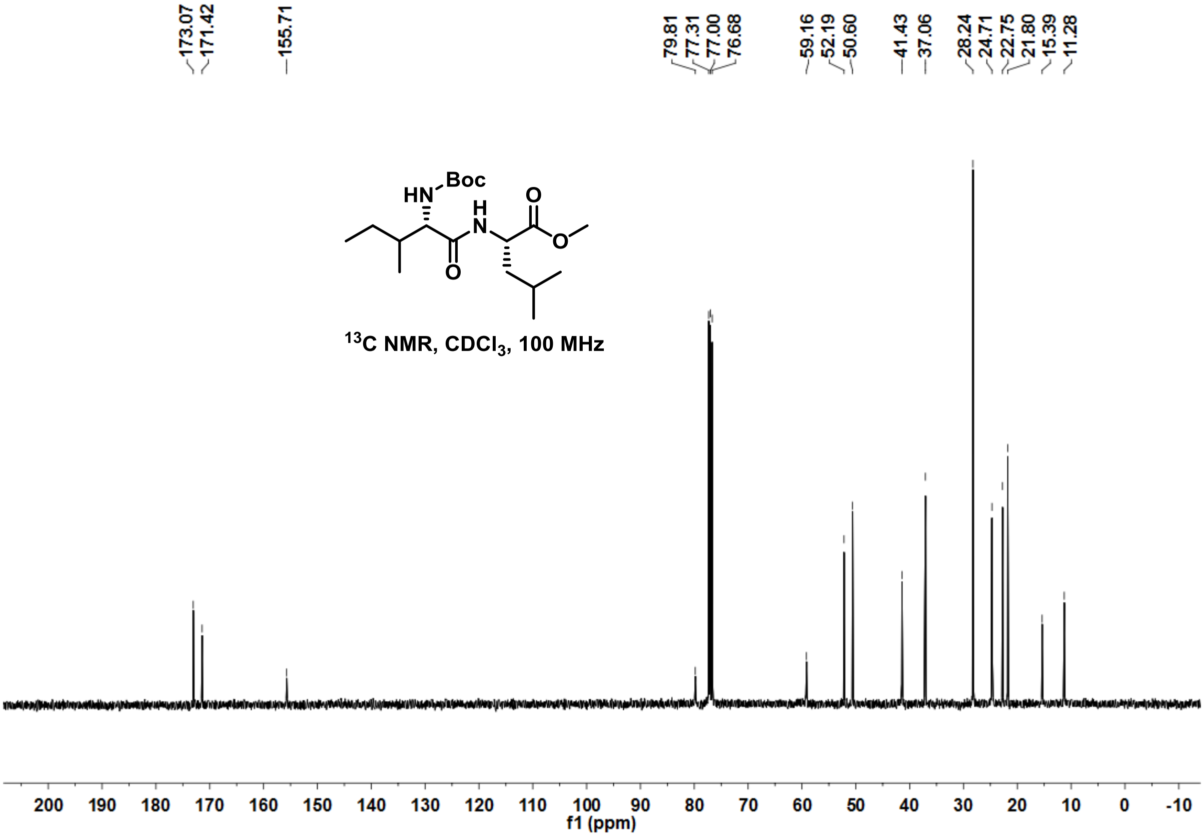

Boc-L-Leu-L-Ile-L-Leu-OMe (8)

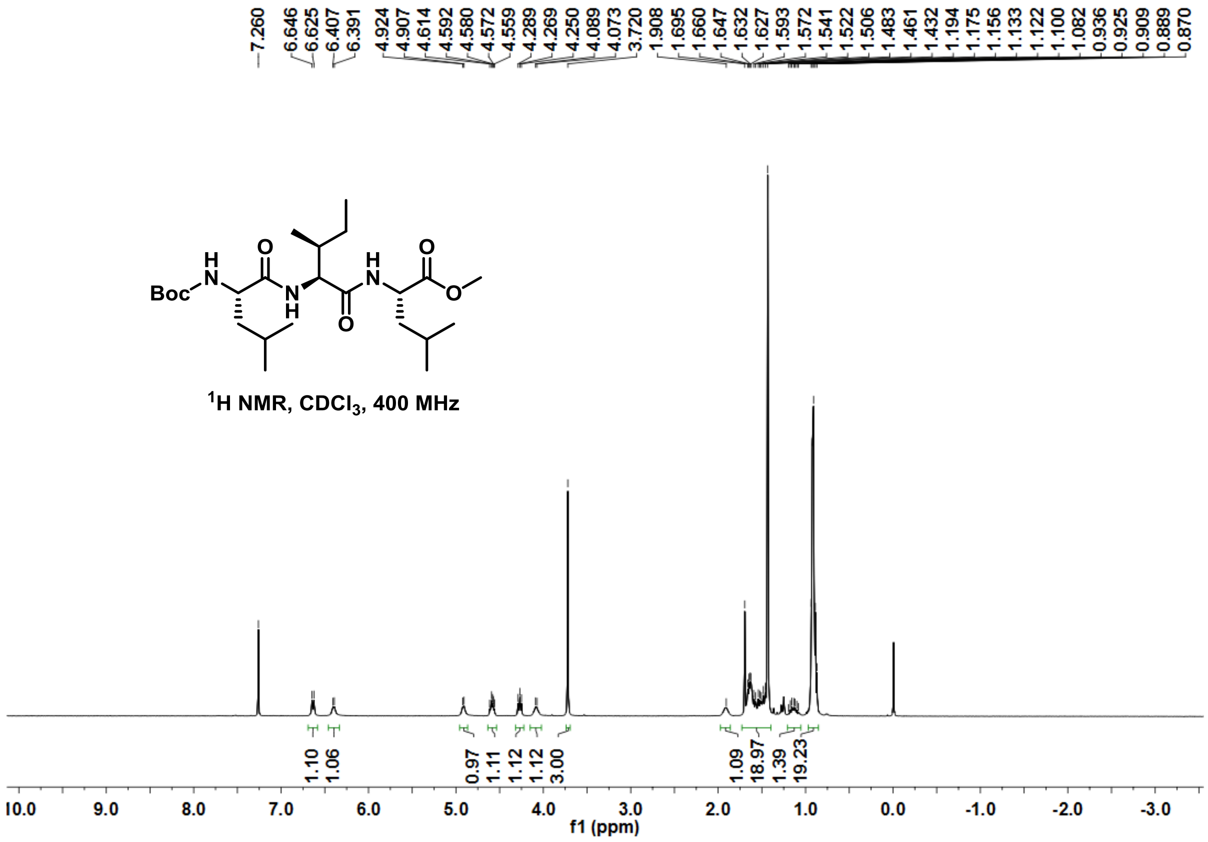

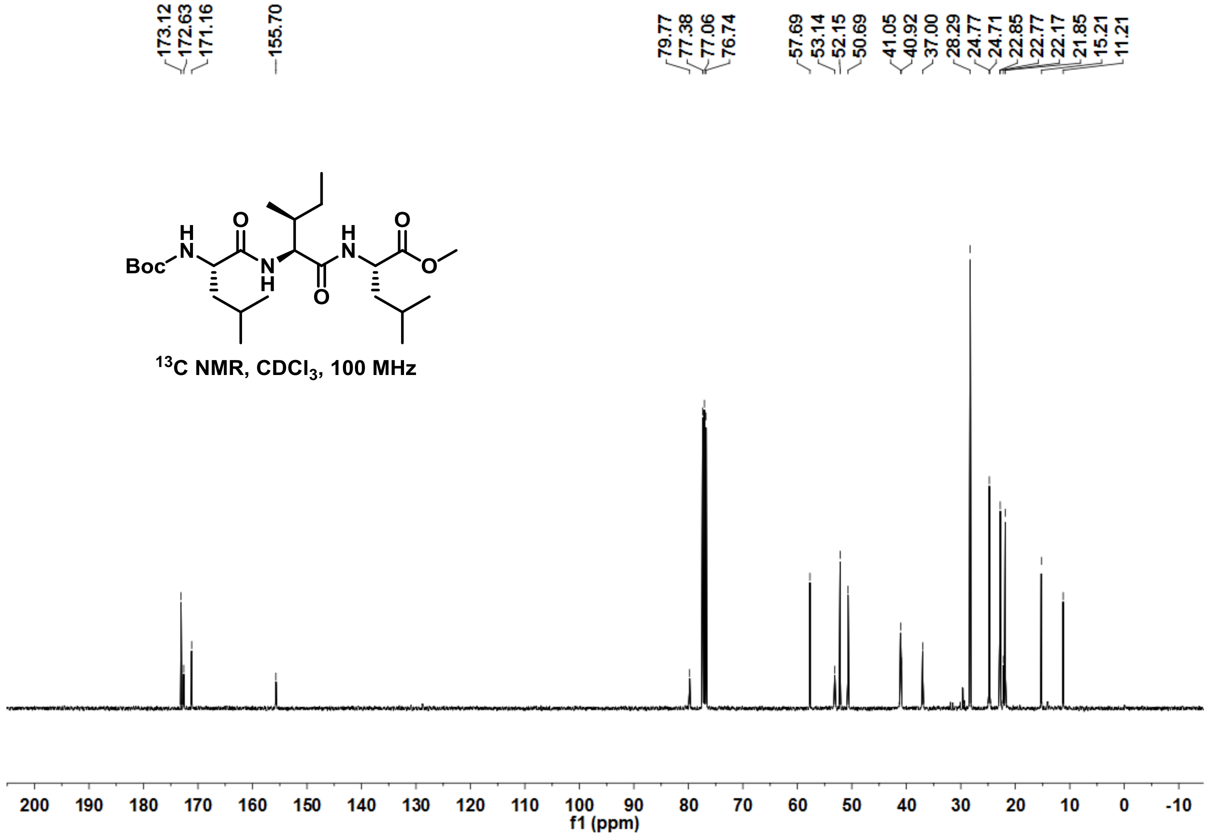

Boc-L-Pro-L-Leu-L-Ile-L-Leu-OMe (9)

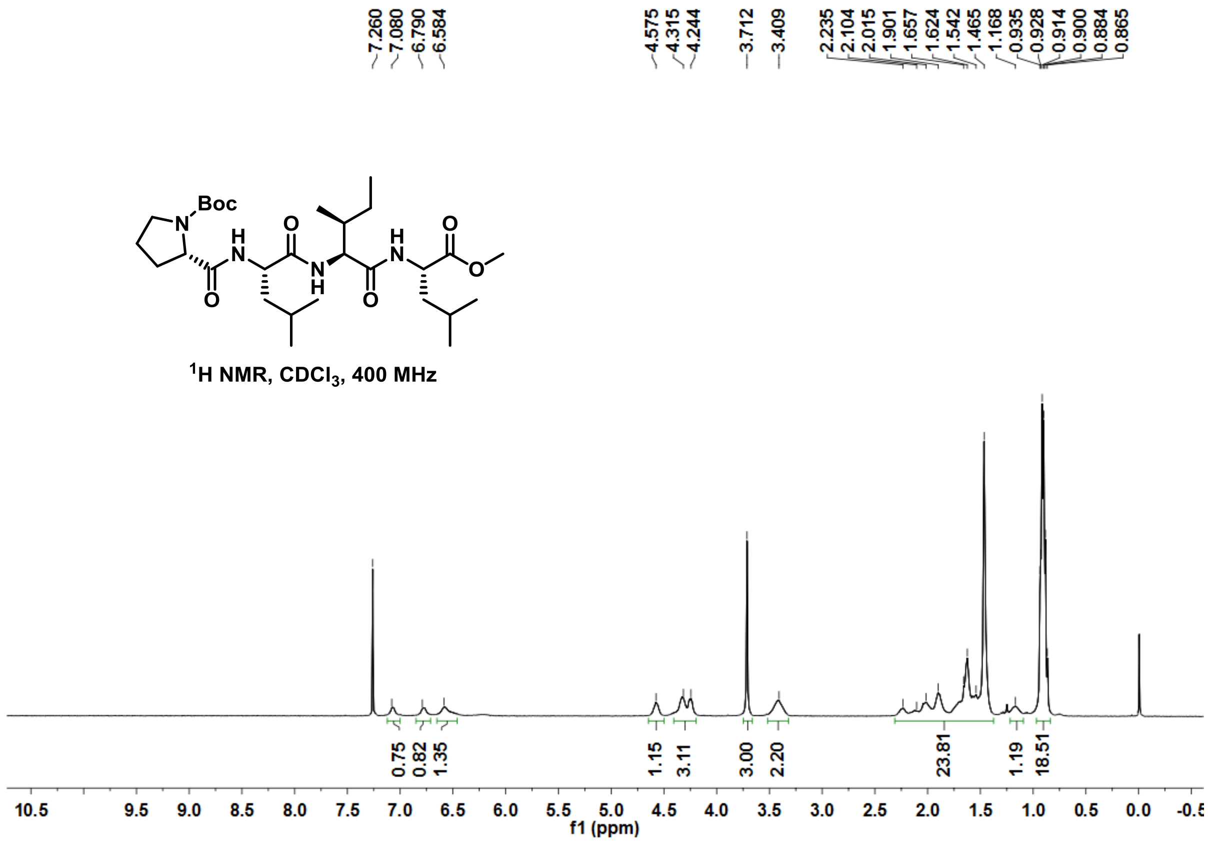

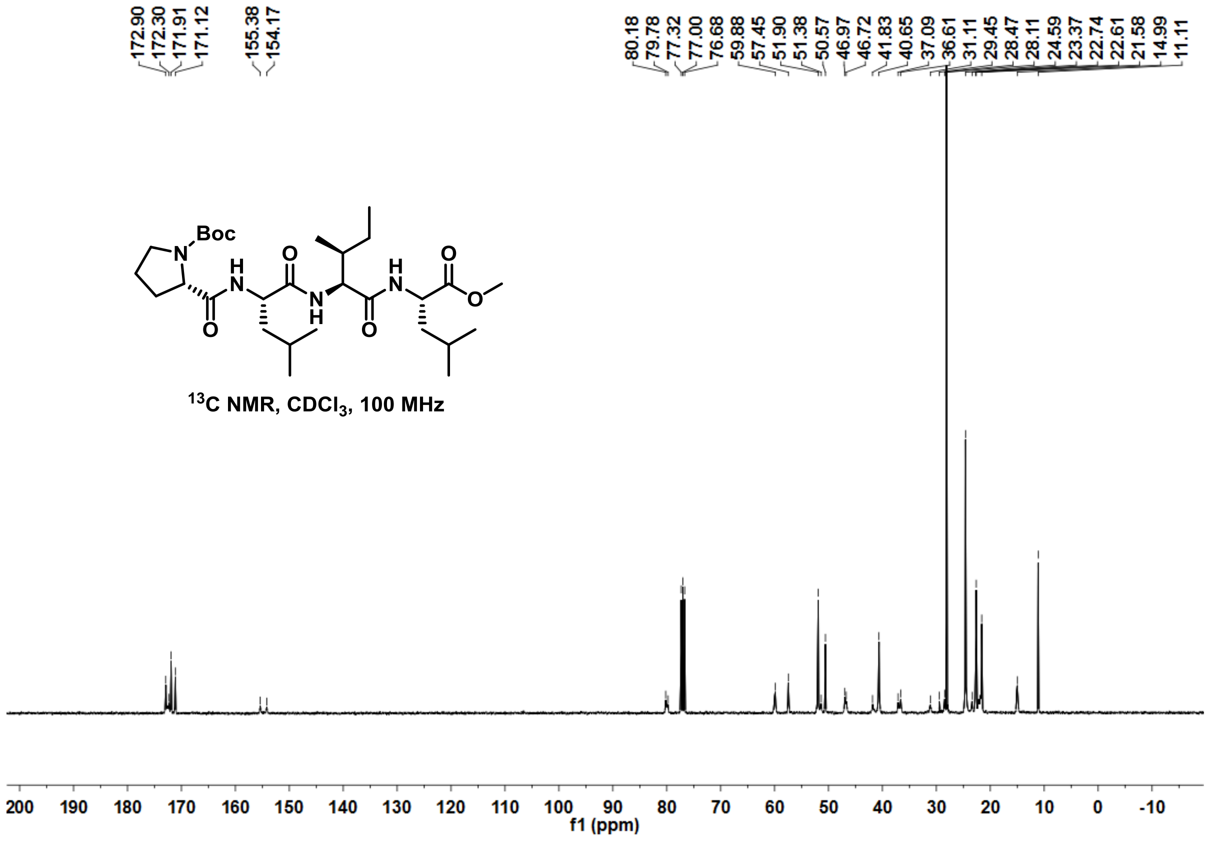

Boc-Gly-Gly-OMe (11)

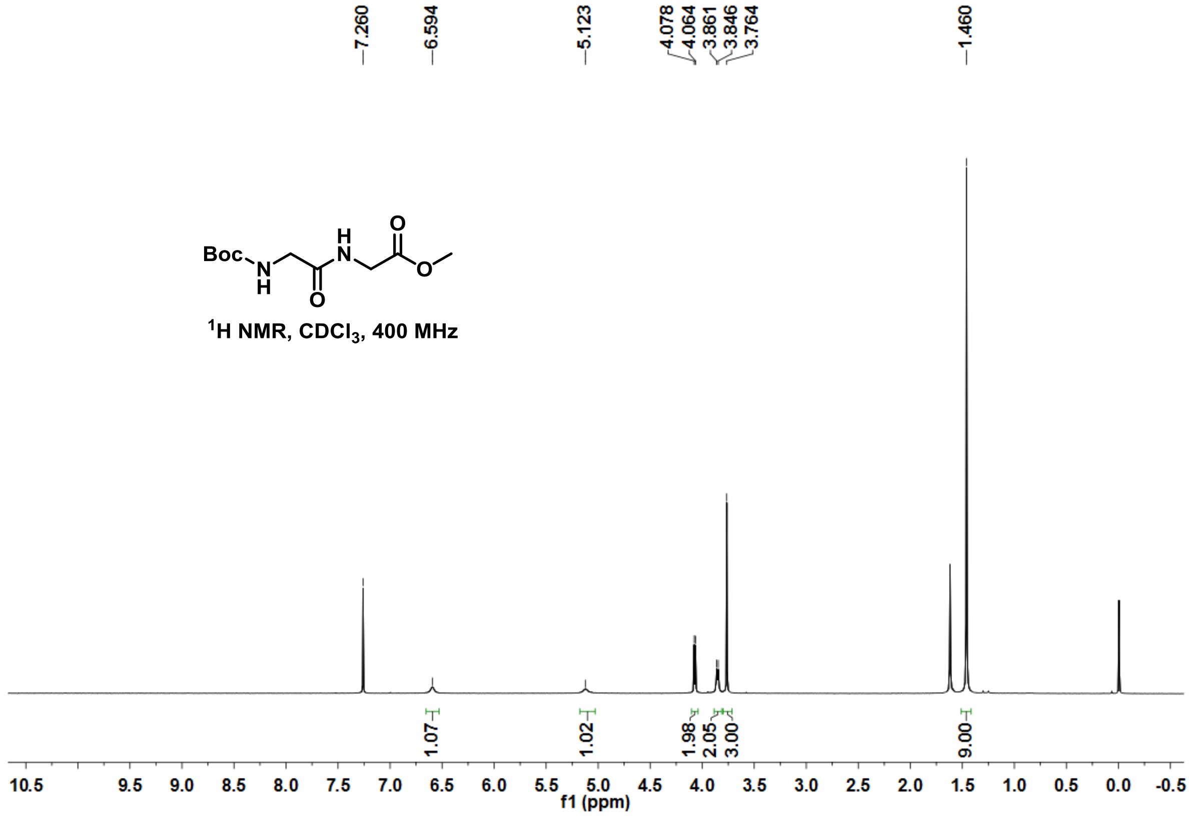

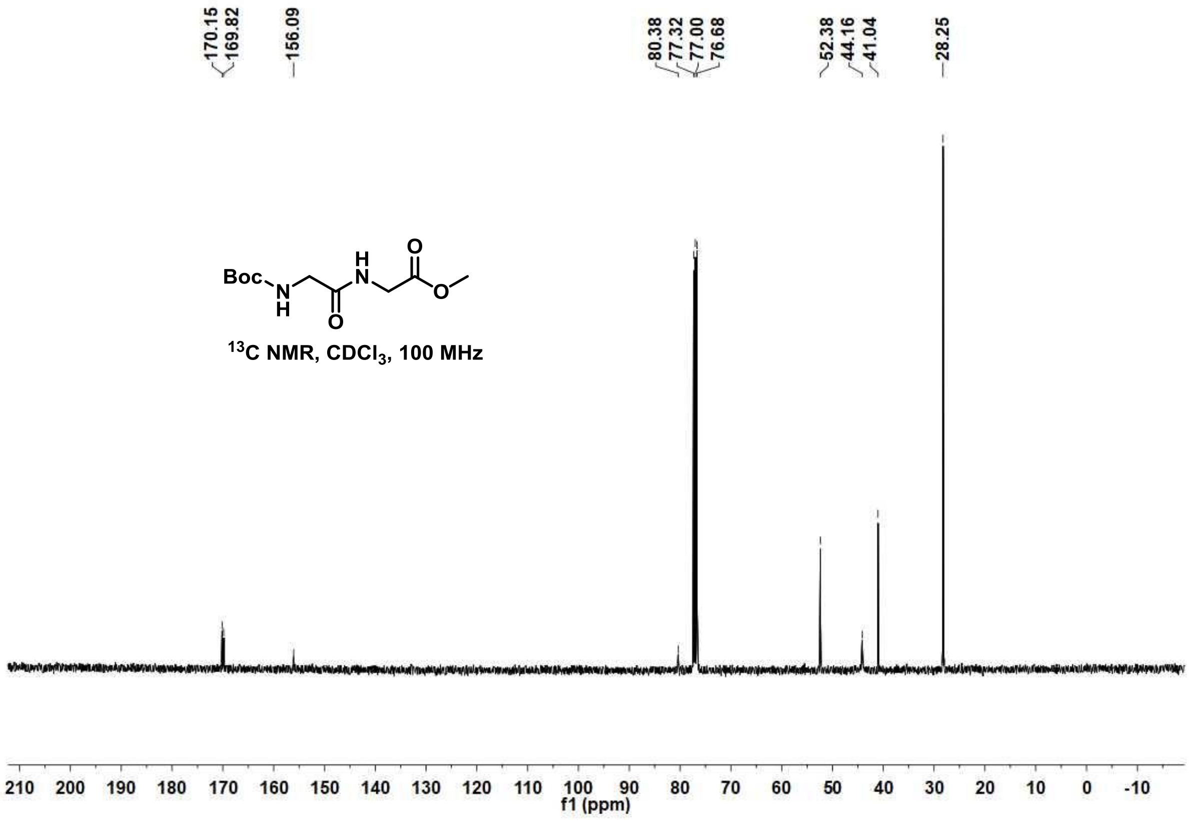

Boc-Gly-Gly-L-Tyr-OMe (12)

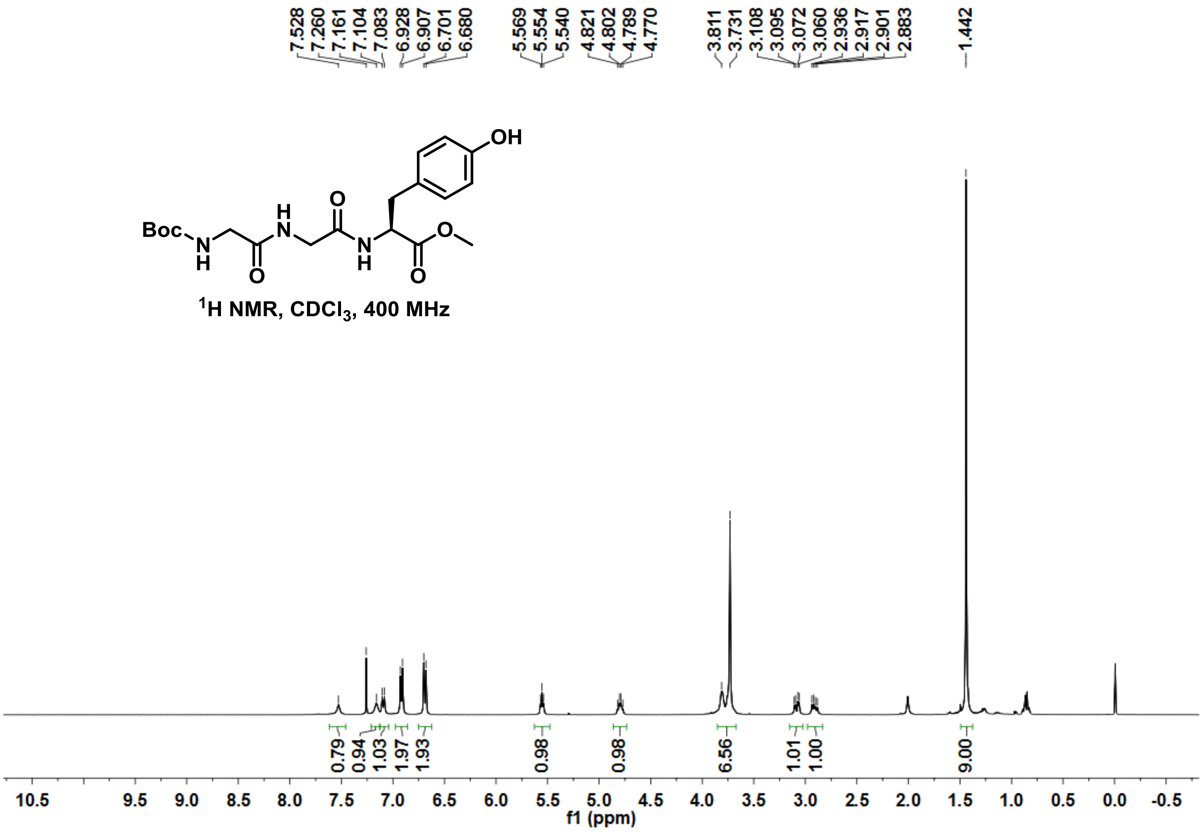

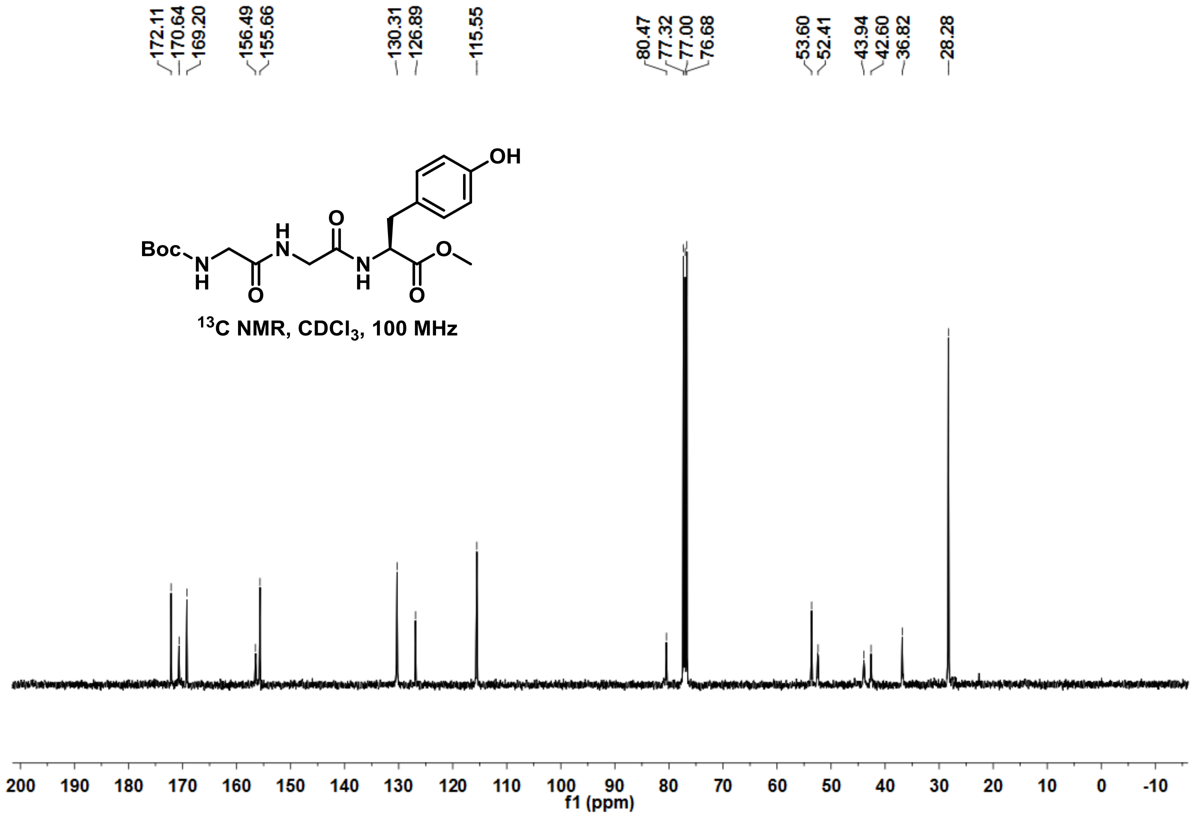

Boc-Gly-Gly-L-Tyr-L-Pro-L-Leu-L-Ile-L-Leu-OMe (14)

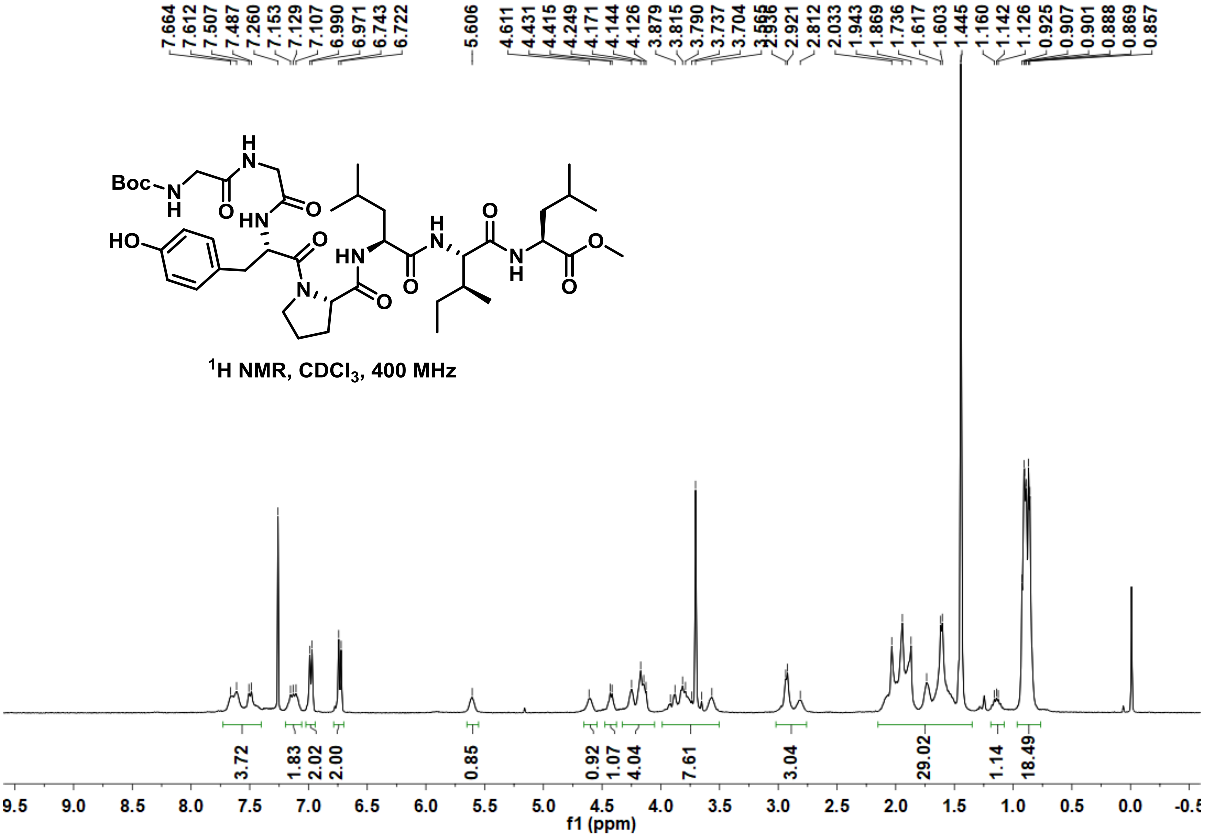

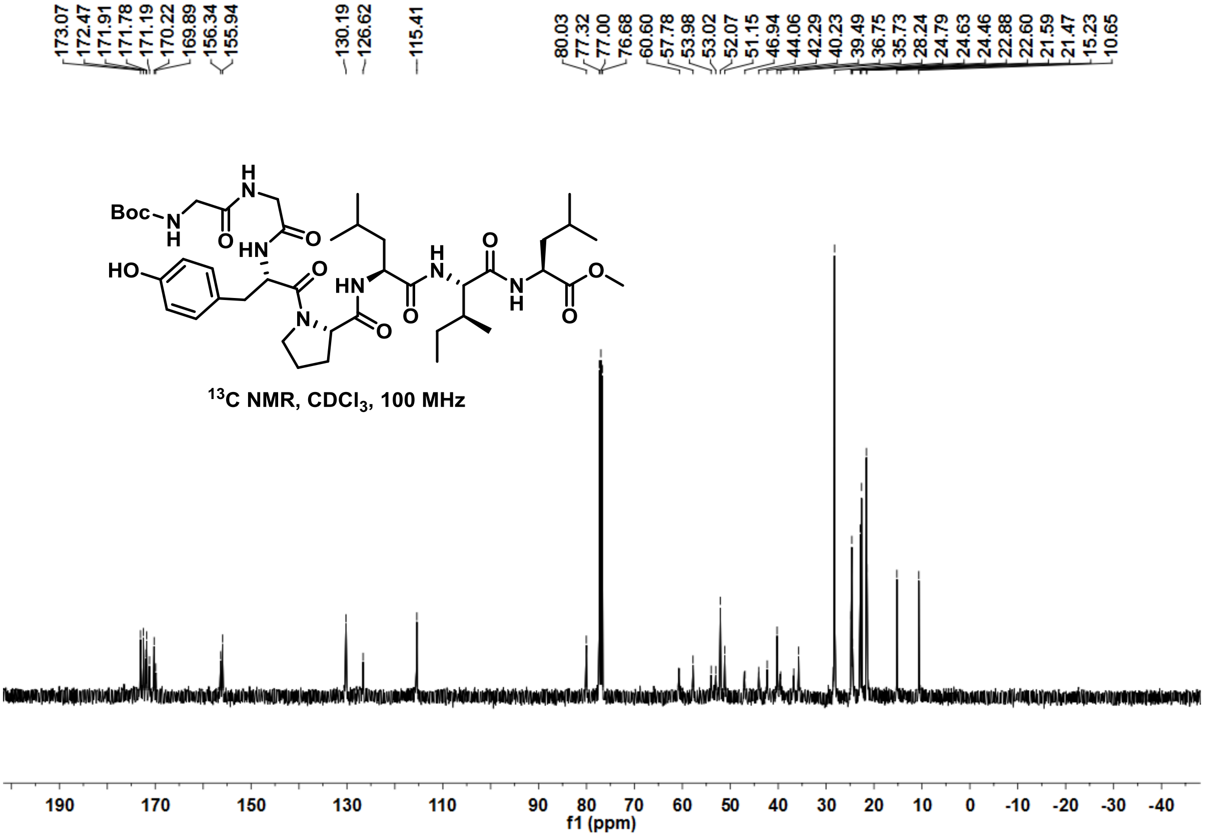

Pseudostellarin D [*Cyclo*(Gly-Gly-Tyr-Pro-Leu-Ile-Leu)]

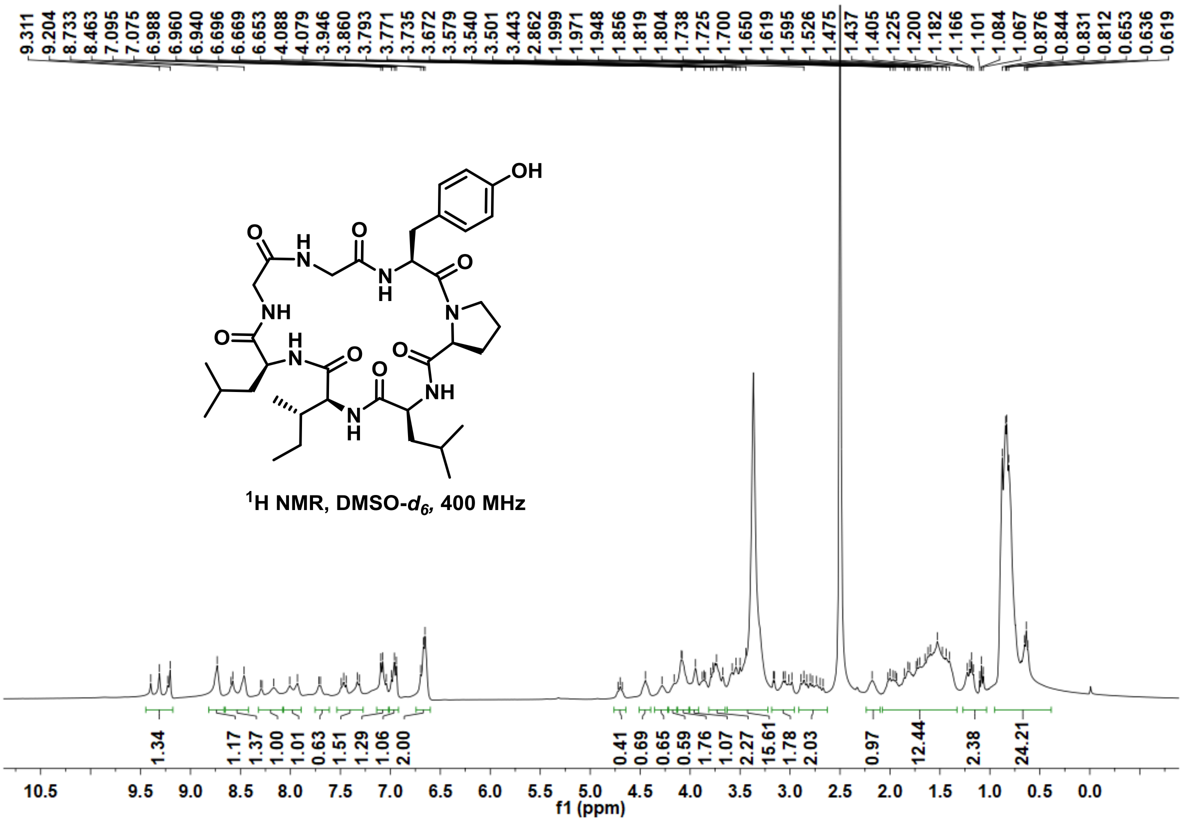

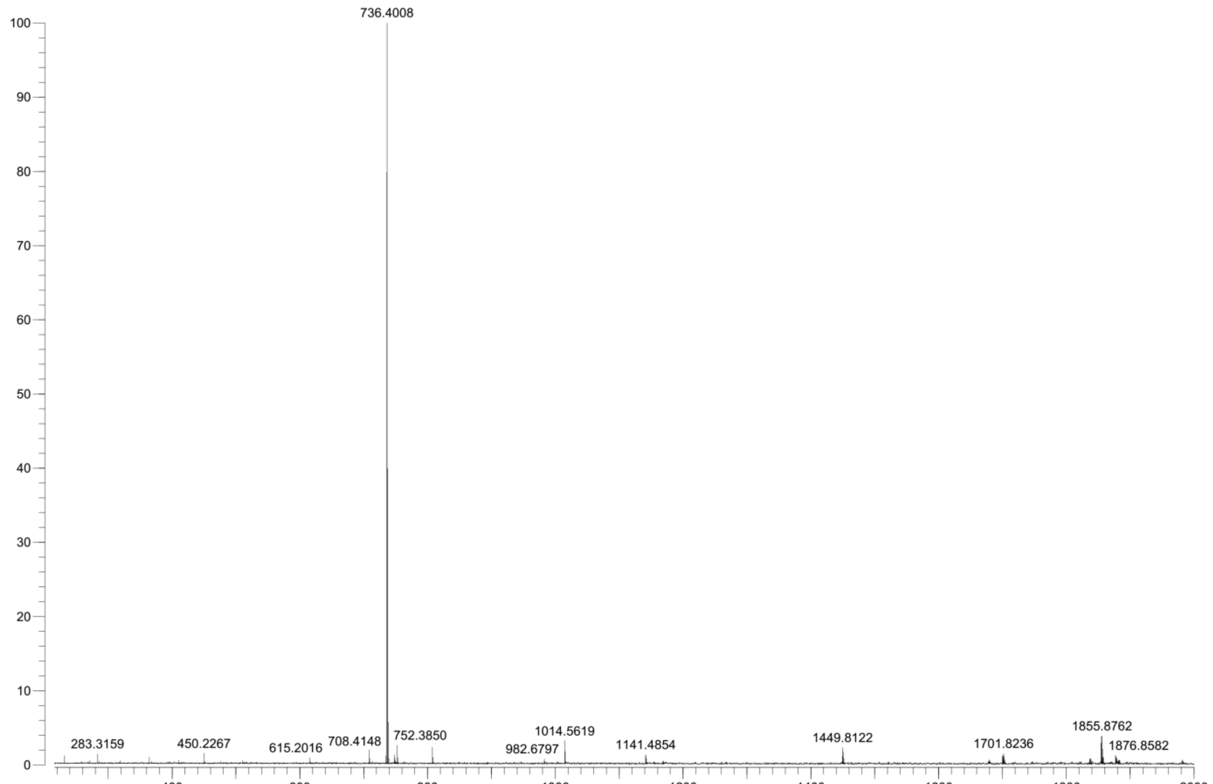

Supplement: File 1 — Experimental procedures and characterization data of all products, copies of 1H, 13C, HPLC, HRMS spectra of some compounds. [file Beilstein_J_Org_Chem-14-1112-s001.pdf]
